# Cytotoxic substituted indolizines as new colchicine site tubulin polymerisation inhibitors

**DOI:** 10.1080/14756366.2020.1801671

**Published:** 2020-08-04

**Authors:** Monica-Cornelia Sardaru, Anda Mihaela Craciun, Cristina-Maria Al Matarneh, Isabela Andreea Sandu, Roxana Maria Amarandi, Lacramioara Popovici, Catalina Ionica Ciobanu, Dragos Peptanariu, Mariana Pinteala, Ionel I. Mangalagiu, Ramona Danac

**Affiliations:** a Department of Chemistry, Faculty of Chemistry, “Al. I. Cuza” University of Iasi, Iași, Romania; b “Petru Poni” Institute of Macromolecular Chemistry of Romanian Academy, Iași, Romania; c TRANSCEND Research Center, Regional Institute of Oncology, Iași, Romania; d Research Department, Faculty of Chemistry, “Al. I. Cuza” University of Iasi, Iași, Romania

**Keywords:** Indolizine, anticancer, tubulin polymerisation inhibitors, Phenstatin, pyridyl

## Abstract

A potential microtubule destabilising series of new indolizine derivatives was synthesised and tested for their anticancer activity against a panel of 60 human cancer cell lines. Compounds **11a**, **11b**, **15a**, and **15j** showed a broad spectrum of growth inhibitory activity against cancer cell lines representing leukaemia, melanoma and cancer of lung, colon, central nervous system, ovary, kidney, breast, and prostate. Among them, compound **11a** was distinguishable by its excellent cytostatic activity, showing GI_50_ values in the range of 10–100 nM on 43 cell lines. The less potent compounds **15a** and **15j** in terms of GI_50_ values showed a high cytotoxic effect against tested colon cancer, CNS cancer, renal cancer and melanoma cell lines and only on few cell lines from other types of cancer. *In vitro* assaying revealed tubulin polymerisation inhibition by all active compounds. Molecular docking showed good complementarity of active compounds with the colchicine binding site of tubulin.

## Graphical Abstract

**Figure:**
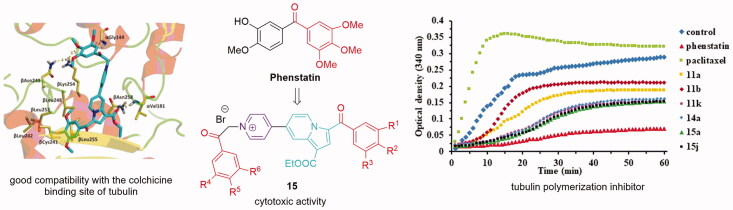

## Introduction

Recognised as key dynamic structural components in cells, microtubules play an important role in cellular shape organisation, intracellular movement, cell division, and mitosis^1^^,^^2^. Thus, they have been considered an attractive target for the development of new antiproliferative agents in the past few years^3–7^.

Commonly, agents targeting tubulin are divided function of the site they interact with tubulin. The three major sites of tubulin are: the paclitaxel binding site (compounds showing microtubule stabilising effects), vinblastine binding site, and colchicine binding site (compounds showing inhibition of tubulin polymerisation)^1^^,^^3–5^^,^^8^. The microtubule-targeting strategy in the drug development field is validated by the use of microtubule-targeting agents such as paclitaxel, docetaxel, vinblastine, colchicine, combretastatin A-4 (CA-4), nocodazole, and many others in cancer chemotherapy^1^^,^^8–10^. However, these compounds also present many drawbacks (high toxicity that induces many side effects, low oral bioavailability, and development of drug resistance) that limit their efficiency^11–13^. Therefore, there is a huge demand of novel antimitotic agents to overcome the abovementioned inconveniences.

**Figure 1.**
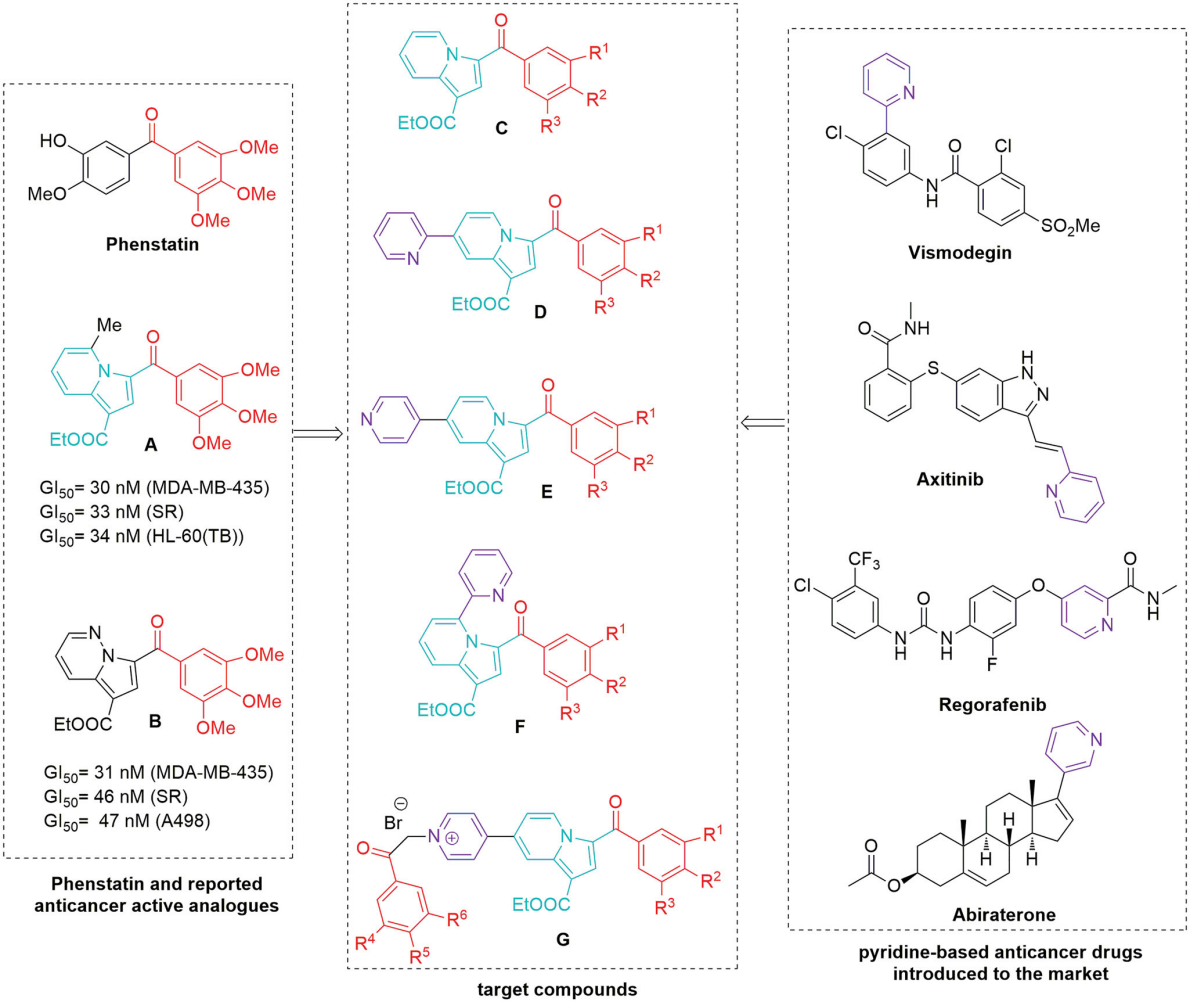
Design in the series of the target indolizine derivatives.

Among the large number of microtubule-targeting agents with diverse scaffolds investigated during last decades^1^^,^^2^^,^^11^, Phenstatin (Figure 1) stands out as one of the simplest molecules that significantly inhibit tubulin polymerisation by binding to the colchicine site of tubulin^14^^,^^15^. Phenstatin is also known for its outstanding antitumor activities on a wide variety of human cancer cells^14^^,^^15^. Its biological properties are comparable to CA-4, currently investigated in clinical trials^16^, but in contrast to CA-4, Phenstatin has a greater pharmacological potential due to improved metabolic stability and requires an easier synthesis for large-scale production^17^. In the process of drug discovery, this kind of compounds are lead scaffolds for the development of improved bioactive analogues, and Phenstatin continues to be a source of inspiration for researchers in designing new potential anticancer drugs^18–25^.

To increase the structural diversity, the combination of various types of bioactive moieties and rings has become a practical strategy in the field of medicinal chemistry. Thus, our study was inspired by several reported anticancer active Phenstatin analogues having the 3′-hydroxy-4′-methoxyphenyl ring replaced with an indolizine moiety^21^ (compound A, Figure 1) or a pyrrolo[1,2-*b*]pyridazine group^24^ (compound B, Figure 1).

At the same time, the pyridine ring, present in many natural products and even genetic material, has been noted for its role in many biological processes as well as in cancer pathogenesis, which makes it a privileged scaffold in anticancer agents discovery^26^. Thus, there are reports of a variety of monosubstituted pyridines with cytotoxic activity^26–29^, and there are also several pyridyl-containing drugs introduced on the market for their antitumor properties^26^ (Figure 1, right).

In our continuous efforts^22^^,^^24^ to discover more effective microtubule destabilising agents, we have applied a structural combination strategy to design and synthesise a new series of indolizine-based Phenstatin analogues and evaluated their anticancer activity. Thus, we considered unsubstituted indolizines (at the pyridine ring) (compounds C, Figure 1), as well as several pyridyl-substituted indolizines (compounds D, E, and F, Figure 1) in order to investigate a possible beneficial influence of this group to the binding properties of generated compounds due to the lone pair electrons in this moiety. In order to increase biological activity, we also designed an indolizine series that contains two 3,4,5-trimethoxybenzoyl groups (compounds G, Figure 1). Furthermore, we modified the 3,4,5-trimethoxyphenyl ring of Phenstatin for each series, replacing it with either a 3,5-dimethoxyphenyl, 3,4-dimethoxyphenyl, or a 4-bromophenyl ring. The structure–activity relationships (SARs), effects on tubulin assembly and theoretical binding interactions were also explored in our study.

## Materials and methods

### Chemistry

All commercially available reagents and solvents employed were used without further purification. Melting points were recorded on a A. Krüss Optronic Melting Point Meter KSPI and are uncorrected. Analytical thin-layer chromatography was performed with commercial silica gel plates 60 F254 (Merck, Darmstadt, Germany) and visualised with UV light (*λ*_max_=254 or 365 nm). The NMR spectra were recorded on an Avance III 500 MHz spectrometer (Bruker, Vienna, Austria) operating at 500 MHz for ^1^H and 125 MHz for ^13^C. Chemical shifts were reported in delta (*δ*) units, part per million (ppm), and coupling constants (*J*) in Hz. The following abbreviations were used to designate chemical shift multiplicities: s = singlet, d = doublet, t = triplet, q = quartet, m = multiplet, and bs = broad singlet. Infrared (IR) data were recorded as films on potassium bromide (KBr) pellets on an FT-IR (Shimadzu, Kyoto, Japan) Prestige 8400s spectrophotometer or a Jasco 660 *plus* FTIR spectrophotometer. Analyses indicated by the symbols of the elements or functions were within ±0.4% of the theoretical values.

#### General procedure for synthesis of monoquaternary salts 8a–l and 13a–d

The corresponding heterocycle (pyridine **1**, 4,4′-bipyridine **2**, 2,4′-bipyridine **3**, or 2,2′-bipyridine **12**) (1 mmol, 1 equiv.) was dissolved in 5–7 ml acetone. Then, reactive halide (2-bromo-1-(3,4,5-trimethoxyphenyl)ethanone **4**, 2-bromo-1-(3,5-dimethoxyphenyl) ethanone **5**, 2-bromo-1-(3,4-dimethoxyphenyl) ethanone **6**, or 2-bromo-1-(4-bromophenyl) ethanone **7**) (1.1 mmol, 1.1 equiv.) was added and the resulted mixture was stirred overnight at room temperature (r.t.). The formed precipitate was filtered and washed with diethyl ether to give the desired product which was used in the next reaction without any further purification.

#### General procedure for preparation of compounds 11a–l and 14a–d

The cycloimmonium salt (**8a–l** and **13a–d)** (1 mmol, 1 equiv.) and ethyl propiolate (1.1 mmol, 1.1 equiv.) were added to dichloromethane (DCM) and the obtained suspension was stirred at r.t. Then, a solution of triethylamine (TEA) (3 mmol, 3 equiv.) in DCM (3 ml) was added drop-wise over 1 h (magnetic stirring) and the resulting mixture was then stirred overnight at r.t. Methanol (10 ml) was added and the resulting solid was collected by filtration and washed with 5 ml methanol. The product was then purified by crystallisation from DCM/methanol (1/1, v/v) and/or column chromatography using DCM/methanol (99.5/0.5, v/v).

#### General procedure for preparation of compounds 15a–j

The monoindolizine **11** (1 mmol, 1 equiv.) and reactive halide derivative (**4**, **5**, or **6**) (2 mmol, 2 equiv.) were suspended in acetone (10 ml) and the resulted reaction mixture was refluxed under magnetical stirring overnight. The resulting precipitate was collected by filtration and then washed with acetone. Compounds **15a–i** were further purified by crystallisation from chloroform/methanol (1:1, v/v).

*Ethyl 3-(3,4,5-trimethoxybenzoyl)indolizine-1-carboxylate (***11a***).* Beige solid, yield 51%, mp 197–198 °C. IR *ν* (cm^−1^): 1701, 1622, 1584, 1526, 1485, 1350, 1223, 1202, 1130, 1055. ^1^H NMR (500 MHz, CDCl_3_): *δ* 1.40 (t, *J* = 7.0 Hz, 3H, CH_3_), 3.92 (s, 6H, 2 × OMe), 3.95 (s, 3H, OMe), 4.38 (q, *J* = 7.0 Hz, 2H, CH_2_), 7.08 (s, 2H, H_12_, H_16_), 7.10 (t, *J* = 7.0 Hz, 1H, H_6_), 7.46 (t, *J* = 7.5 Hz, 1H, H_7_), 7.88 (s, 1H, H_2_), 8.40 (d, *J* = 8.5 Hz, 1H, H_8_), 9.91 (d, *J* = 6.5 Hz, 1H, H_5_). ^13^C NMR (125 MHz, CDCl_3_): *δ* 14.7 CH_3_, 56.52 × OMe, 60.3 CH_2_, 61.2 OMe, 106.5 C_1_, 107.8 C_12_, C_16_, 115.4 C_6_, 119.7 C_8_, 122.5 C_3_, 127.8 C_7_, 128.9 C_2_, 129.3 C_5_, 135.2 C_11_, 140.1 C_9_, 141.4 C_14_, 153.2 C_13_, C_15_, 164.2 COO, 184.8 C_10_. Anal. Calcd. for C_21_H_21_NO_6_: C, 65.79; H, 5.52; N, 3.65. Found: C, 65.74; H, 5.52; N, 3.70.

*Ethyl 3-(3,5-dimethoxybenzoyl)indolizine-1-carboxylate (***11b***).* Beige solid, yield 50%, mp 178–180 °C. IR *ν* (cm^−1^): 1699, 1628, 1593, 1528, 1458, 1356, 1200, 1155, 1045. ^1^H NMR (500 MHz, CDCl_3_): *δ* 1.40 (t, *J* = 7.0 Hz, 3H, CH_3_), 3.86 (s, 6H, 2 × OMe), 4.38 (q, *J* = 7.0 Hz, 2H, CH_2_), 6.66 (s, 1H, H_14_), 6.93 (s, 2H, H_12_, H_16_), 7.09 (t, *J* = 7.0 Hz, 1H, H_6_), 7.46 (t, *J* = 7.5 Hz, 1H, H_7_), 7.87 (s, 1H, H_2_), 8.40 (d, *J* = 8.0 Hz, 1H, H_8_), 9.95 (d, *J* = 7.0 Hz, 1H, H_5_). ^13^C NMR (125 MHz, CDCl_3_): *δ* 14.7 CH_3_, 55.82 × OMe, 60.3 CH_2_, 103.8 C_14_, 106.5 C_1_, 107.0 C_12_, C_16_, 115.5 C_6_, 119.7 C_8_, 122.5 C_3_, 127.9 C_7_, 129.2 C_5_, 129.4 C_2_, 140.2 C_9_, 142.0 C_11_, 160.8 C_13_, C_15_, 164.2 COO, 185.3 C_10_. Anal. Calcd. for C_20_H_19_NO_5_: C, 67.98; H, 5.42; N, 3.96. Found: C, 68.00; H, 5.40; N, 3.90.

*Ethyl 3-(3,4-dimethoxybenzoyl)indolizine-1-carboxylate (***11c***).* Beige solid, yield 56%, mp 196–198 °C. IR *ν* (cm^−1^): 1703, 1610, 1514, 1483, 1261, 1211, 1138, 1047; ^1^H NMR (500 MHz, CDCl_3_): *δ* 1.40 (t, *J* = 7.0 Hz, 3H, CH_3_), 3.96 (s, 3H, OMe), 3.98 (s, 3H, OMe), 4.38 (q, *J* = 7.0 Hz, 2H, CH_2_), 6.96 (d, *J* = 8.5 Hz, 1H, H_15_), 7.07 (t, *J* = 7.0 Hz, 1H, H_6_), 7.41–7.45 (overlapped signals, 2H, H_7_, H_12_), 7.48 (d, *J* = 8.0 Hz, 1H, H_16_), 7.86 (s, 1H, H_2_), 8.39 (d, *J* = 8.5 Hz, 1H, H_8_), 9.88 (d, *J* = 7.0 Hz, 1H, H_5_). ^13^C NMR (125 MHz, CDCl_3_): *δ* 14.7 CH_3_, 56.2 OMe, 56.3 OMe, 60.2 CH_2_, 106.2 C_1_, 110.3 C_15_, 111.8 C_12_, 115.2 C_6_, 119.7 C_8_, 122.5 C_3_, 123.6 C_16_, 127.5 C_7_, 128.5 C_2_, 129.2 C_5_, 132.6 C_11_, 139.9 C_9_, 149.2 C_13_, 153.4 C_14_, 164.3 COO, 184.6 C_10_. Anal. Calcd. for C_20_H_19_NO_5_: C, 67.98; H, 5.42; N, 3.96. Found: C, 67.64; H, 5.40; N, 3.93.

*Ethyl 3-(4-bromobenzoyl)indolizine-1-carboxylate (***11d***)*^30^. Beige solid, yield 50%, mp 130–132 °C. IR *ν* (cm^−1^): 3086, 2978, 1697, 1612, 1522, 1479, 1339, 1219, 1140, 1043. ^1^H NMR (500 MHz, CDCl_3_): *δ* 1.41 (t, *J* = 7.2 Hz, 3H, CH_3_), 4.38 (q, *J* = 7.2 Hz, 2H, CH_2_), 7.10 (dt, *J* = 6.8; 1.2 Hz, 1H, H_6_), 7.47 (m, 1H, H_7_), 7.65 (d, *J* = 8.4 Hz, 2H, H_12_, H_16_), 7.70 (d, *J* = 8.4 Hz, 2H, H_13_, H_15_), 7.75 (s, 1H, H_2_), 8.40 (d, *J* = 8.8 Hz, 1H, H_8_), 9.93 (d, *J* = 6.8 Hz, 1H, H_5_). ^13^C NMR (125 MHz, CDCl_3_): *δ* 14.5 CH_3_, 60.2 CH_2_, 106.5 C_1_, 115.4 C_6_, 119.5 C_8_, 122.1 C_3_, 126.2 C_14_, 127.9 C_7_, 128.8 C_2_, 129.1 C_5_, 130.5 C_13_, C_15_, 131.6 C_12_, C_16_, 140.0 C_9_, 138.6 C_11_, 163.9 COO, 184.1 C_10_. Anal. Calcd. for C_18_H_14_BrNO_3_: C, 58.08; H, 3.79; N, 3.76. Found: C, 58.04; H, 3,80; N, 3.80.

*Ethyl 7-(pyridin-4-yl)-3-(3,4,5-trimethoxybenzoyl)indolizine-1-carboxylate (***11e***).* Beige solid, yield 70%, mp 194–196 °C. IR *ν* (cm^−1^): 1701, 1622, 1206, 1580, 1528, 1471, 1352, 1206, 1132, 1051. ^1^H NMR (500 MHz, CDCl_3_): *δ* 1.42 (as, 3H, CH_3_), 3.93 (s, 6H, 2 x OMe), 3.96 (s, 3H, OMe), 4.41 (bs, 2H, CH_2_), 7.10 (s, 2H, H_12_, H_16_), 7.37 (as, 1H, H_6_), 7.69 (as, 2H, 2 × H_py_), 7.91 (s, 1H, H_2_), 8.75 (overlapped signals, 3H, 2 × H_py_, H_8_), 9.96 (as, 1H, H_5_). ^13^C NMR (125 MHz, CDCl_3_): *δ* 14.7 CH_3_, 56.52 × OMe, 60.5 CH_2_, 61.2 OMe, 107.6 C_1_, 106.9 C_12_, C_16_, 113.7 C_6_, 117.3 C_8_, 121.62 × CH_py_, 122.8 C_3_, 129.0 C_2_, 129.6 C_5_, 134.9 C_14_, 137.0 C_7_, 139.9 C_9_, 141.6 C_11_, 145.3 C_py_, 150.82 × CH_py_, 153.2 C_13_, C_15_, 164.1 COO, 184.9 C_10_. Anal. Calcd. for C_26_H_24_N_2_O_6_: C, 67.82; H, 5.25; N, 6.08. Found: C, 67.84; H, 5.20; N, 6.09.

*Ethyl 3-(3,5-dimethoxybenzoyl)-7-(pyridin-4-yl)indolizine-1-carboxylate (***11f***).* Beige solid, yield 55%, mp 208–210 °C. IR *ν* (cm^−1^): 1717, 1684, 1595, 1514, 1468, 1362, 1246, 1207, 1159, 1045. ^1^H NMR (500 MHz, CDCl_3_): *δ* 1.42 (t, *J* = 7.0 Hz, 3H, CH_3_), 3.86 (s, 6H, 2 × OMe), 4.40 (q, *J* = 7.0 Hz, 2H, CH_2_), 6.68 (s, 1H, H_14_), 6.95 (s, 2H, H_12_, H_16_), 7.36 (ad, *J* = 7.0 Hz, 1H, H_6_), 7.67 (as, 2H, 2 × H_py_), 7.90 (s, 1H, H_2_), 8.74 (overlapped signals, 3H, 2 × H_py_, H_8_), 9.99 (ad, *J* = 7.0 Hz, 1H, H_5_). ^13^C NMR (125 MHz, CDCl_3_): *δ* 14.7 CH_3_, 55.8 2 × OMe, 60.4 CH_2_, 104.0 C_14_, 107.1 C_12_, C_16_, 107.6 C_1_, 113.8 C_6_, 117.3 C_8_, 121.4 2 × CH_py_, 122.8 C_3_, 129.4 C_2_, 129.7 C_5_, 137.0 C_7_, 139.9 C_9_, 141.6 C_11_, 145.3 C_py_, 150.82 × CH_py_, 160.8 C_13_, C_15_, 164.0 COO, 184.4 C_10_. Anal. Calcd. for C_25_H_22_N_2_O_5_: C, 69.76; H, 5.15; N, 6.51. Found: C, 69.74; H, 5.10; N, 6.50.

*Ethyl 3-(3,4-dimethoxybenzoyl)-7-(pyridin-4-yl)indolizine-1-carboxylate (***11g***).* Beige solid, yield 56%, mp 192–195 °C. IR *ν* (cm^−1^): 3079, 2979, 1691, 1596, 1514, 1347, 1266, 1219, 1141, 1020. ^1^H NMR (500 MHz, CDCl_3_): *δ* 1.42 (t, *J* = 7.0 Hz, 3H, CH_3_), 3.97 (s, 3H, OMe), 3.99 (s, 3H, OMe), 4.41 (q, *J* = 7.0 Hz, 2H, CH_2_), 6.98 (d, *J* = 8.5 Hz, 1H, H_15_), 7.35 (d, *J* = 7.0 Hz, 2H, H_6_), 7.45 (s, 1H, H_12_), 7.51 (d, *J* = 7.0 Hz, 1H, H_16_), 7.66 (d, *J* = 5.0 Hz, 2H, 2 × H_py_), 7.89 (s, 1H, H_2_), 8.75 (overlapped signals, 3H, 2 × H_py_, H_8_), 9.93 (d, *J* = 7.0 Hz, 1H, H_5_). ^13^C NMR (125 MHz, CDCl_3_): *δ* 14.7 CH_3_, 56.2 OMe, 56.3 OMe, 60.4 CH_2_, 107.3 C_1_, 110.3 C_15_, 111.7 C_12_, 113.5 C_6_, 117.3 C_8_, 121.4 2 × CH_py_, 123.0 C_3_, 123.7 C_16_, 128.7 C_5_, 129.5 C_2_, 132.2 C_11_, 136.6 C_7_, 139.6 C_9_, 145.5 C_py_, 149.3 C_13_, 150.8 2 × CH_py_, 152.6 C_14_, 164.2 COO, 184.7 C_10_; Anal. Calcd. for C_25_H_22_N_2_O_5_: C, 69.76; H, 5.15; N, 6.51. Found: C, 69.72; H, 5.11; N, 6.55.

*Ethyl 3-(4-bromobenzoyl)-7-(pyridin-4-yl)indolizine-1-carboxylate (***11h***).* Yield 65%. All spectral data are in agreement with the literature^31^.

*Ethyl 7-(pyridin-2-yl)-3-(3,4,5-trimethoxybenzoyl)indolizine-1-carboxylate (***11i***).* Yellow solid, yield 41%, mp 202–203 °C. IR *ν* (cm^−1^): 2994, 2926, 1700, 1620, 1580, 1480, 1352, 1204, 1136, 780. ^1^H NMR (500 MHz, CDCl_3_): *δ* 1.43 (t, *J* = 7.0 Hz, 3H, CH_3_), 3.94 (s, 6H, 2 × OMe), 3.97 (s, 3H, OMe), 4.42 (q, *J* = 7.0 Hz, 2H, CH_2_), 7.11 (s, 2H, H_12_, H_16_), 7.35 (dd, *J* = 7.5; 5.0 Hz, 1H, H_py_), 7.85 (dt, *J* = 8.0; 2.0 Hz, 1H, H_py_), 7.90 (s, 1H, H_2_), 7.92 (dd, *J* = 7.5; 2.0 Hz, 1H, H_6_), 7.97 (d, *J* = 8.0 Hz, 1H, H_py_), 8.77 (d, *J* = 4.5 Hz, 1H, H_py_), 9.01 (bs, 1H, H_8_), 9.95 (d, *J* = 7.0 Hz, 1H, H_5_). ^13^C NMR (125 MHz, CDCl_3_): *δ* 14.7 CH_3_, 55.62 × OMe, 60.5 CH_2_, 61.2 OMe, 107.0 C_12_, C_16_, 108.0 C_1_, 113.9 C_6_, 118.1 C_8_, 122.5 CH_py_, 123.2 C_3_, 124.3 CH_py_, 128.9 C_2_, 129.6 C_5_, 134.9 C_11_, 138.9 C_7_, 139.5 C_9_, 140.2 CH_py_, 141.7 C_14_, 147.9 CH_py_, 153.2 C_py_, 153.3 C_13_, C_15_, 164.1 COO, 184.9 C_10_. Anal. Calcd. for C_26_H_24_N_2_O_6_: C, 67.82; H, 5.25; N, 6.08. Found: C, 67.85; H, 5.17; N, 6.13.

*Ethyl 3-(3,5-dimethoxybenzoyl)-7-(pyridin-2-yl)indolizine-1-carboxylate (***11j***).* Yellow solid, yield 40%, mp 165–166 °C. IR *ν* (cm^−1^): 2934, 1696, 1587, 1448, 1352, 1209, 1152, 1044, 774. ^1^H NMR (500 MHz, CDCl_3_): *δ* 1.43 (t, 3H, *J* = 7.0 Hz, CH_3_), 3.87 (s, 6H, 2 × OMe), 4.41 (q, 2H, *J* = 7.0 Hz, CH_2_), 6.68 (t, *J* = 2.5 Hz, 1H, H_14_), 6.96 (d, *J* = 2.5 Hz, 2H, H_12_, H_16_), 7.34 (dd, *J* = 7.0; 5.0 Hz, 1H, H_py_), 7.84 (dt, *J* = 7.5; 2.0 Hz, 1H, H_py_), 7.89 (s, 1H, H_2_), 7.92 (dd, *J* = 7.5; 2.0 Hz, 1H, H_6_), 7.97 (d, *J* = 8.0 Hz, 1H, H_py_), 8.77 (d, *J* = 4.5 Hz, 1H, H_py_), 9.00 (d, *J* = 1.0 Hz, 1H, H_8_), 9.99 (d, *J* = 7.5 Hz, 1H, H_5_). ^13^C NMR (125 MHz, CDCl_3_): *δ* 14.7 CH_3_, 55.82 × OMe, 60.4 CH_2_, 103.9 C_14_, 107.0 C_12_, C_16_, 107.5 C_1_, 114.0 C_6_, 116.8 C_8_, 121.0 CH_py_, 122.8 C_3_, 123.6 CH_py_, 129.4 C_2_, 129.5 C_5_, 137.3 CH_py_, 138.6 C_7_, 140.3 C_9_, 141.9 C_11_, 150.1 CH_py_, 154.4 C_py_, 160.8 C_13_, C_15_, 164.2 COO, 185.6 C_10_. Anal. Calcd. for C_25_H_22_N_2_O_5_: C, 69.76; H, 5.15; N, 6.51. Found: C, 69.79; H, 5.10; N, 6.53.

*Ethyl 3-(3,4-dimethoxybenzoyl)-7-(pyridin-2-yl)indolizine-1-carboxylate (***11k***).* Yellow solid, yield 44%, mp 199–200 °C. IR *ν* (cm^−1^): 2974, 2931, 1699, 1608, 1517, 1472, 1426, 1347, 1267, 1199, 773. ^1^H NMR (500 MHz, CDCl_3_): *δ* 1.44 (t, *J* = 7.0 Hz, 3H, CH_3_), 3.98 (s, 3H, OMe), 4.00 (s, 3H, OMe), 4.42 (q, *J* = 7.0 Hz, 2H, CH_2_), 6.99 (d, *J* = 8.5 Hz, 1H, H_15_), 7.46 (bs, 1H, H_12_), 7.33 (dd, *J* = 7.0; 5.0 Hz, 1H, H_py_), 7.85 (t, *J* = 7.5 Hz, 1H, H_py_), 7.88 (s, 1H, H_2_), 7.90 (dd, *J* = 7.5; 1.5 Hz, 1H, H_6_), 7.97 (d, *J* = 8.0 Hz, 1H, H_py_), 8.77 (d, *J* = 4.5 Hz,1H, H_py_), 7.52 (dd, *J* = 8.0; 1.5 Hz, 1H, H_16_), 9.00 (bs, 1H, H_8_), 9.93 (d, *J* = 7.5 Hz, 1H, H_5_). ^13^C NMR (125 MHz, CDCl_3_): *δ* 14.7 CH_3_, 56.2 OMe, 56.3 OMe, 60.3 CH_2_, 107.2 C_1_, 110.3 C_15_, 111.8 C_12_, 113.8 C_6_, 116.8 C_8_, 121.0 CH_py_, 123.0 C_3_, 123.5 CH_py_, 123.7 C_16_, 128.8 C_2_, 129.2 C_5_, 132.5 C_11_, 137.2 CH_py_, 138.2 C_7_, 140.0 C_9_, 149.2 C_13_, 150.1 CH_py_, 152.5 C_14_, 154.5 C_py_, 164.3 COO, 184.6 C_10_. Anal. Calcd. for C_25_H_22_N_2_O_5_: C, 69.76; H, 5.15; N, 6.51. Found: C, 69.78; H, 5.09; N, 6.53.

*Ethyl 3-(4-bromobenzoyl)-7-(pyridin-2-yl)indolizine-1-carboxylate (***11l***).* Yellow solid, yield 40%, mp 197–198 °C. IR *ν* (cm^−1^): 2928, 1694, 1620, 1526, 1479, 1342, 1200, 1079. ^1^H NMR (500 MHz, CDCl_3_): *δ* 1.43 (t, *J* = 7.0 Hz, 3H, CH_3_), 4.41 (q, *J* = 7.0 Hz, 2H, CH_2_), 7.34 (dd, *J* = 7.5; 4.5 Hz, 1H, H_py_), 7.68 (d, *J* = 8.0 Hz, 2H, H_12_, H_16_), 7.73 (d, *J* = 8.0 Hz, 2H, H_13_, H_15_), 7.80 (s, 1H, H_2_), 7.84 (dt, *J* = 7.5; 2.0 Hz, 1H, H_py_), 7.94 (dd, *J* = 7.5; 2.0 Hz, 1H, H_6_), 7.97 (d, *J* = 8.0 Hz, 1H, H_py_), 8.77 (d, *J* = 4.5 Hz, 1H, H_py_), 9.01 (d, *J* = 1.0 Hz, 1H, H_8_), 9.98 (d, *J* = 7.5 Hz, 1H, H_5_). ^13^C NMR (125 MHz, CDCl_3_): *δ* 14.7 CH_3_, 60.6 CH_2_, 108.5 C_1_, 114.0 C_6_, 118.3 C_8_, 122.7 CH_py_, 123.0 C_3_, 124.4 CH_py_, 126.8 C_14_, 129.1 C_2_, 129.7 C_5_, 130.7 C_13_, C_15_, 132.0 C_12_, C_16_, 138.5 C_11_, C_7_, 139.5 C_9_, 140.2 CH_py_, 147.6 CH_py_, 153.0 C_py_, 164.0 COO, 184.5 C_10_. Anal. Calcd. for C_23_H_17_BrN_2_O_3_: C, 61.48; H, 3.81; N, 6.23. Found: C, 61.49; H, 3.78; N, 6.26.

*Ethyl 5-(pyridin-2-yl)-3-(3,4,5-trimethoxybenzoyl)indolizine-1-carboxylate (***14a***).* Cream solid, yield 35%, mp 161–163 °C. IR *ν* (cm^−1^): 2984, 1686, 1639, 1585, 1520, 1419, 1350, 1234, 1130, 1059, 752. ^1^H NMR (500 MHz, CDCl_3_): *δ* 1.40 (t, *J* = 7.0 Hz, 3H, CH_3_), 3.87 (s, 6H, 2 × OMe), 3.95 (s, 3H, OMe), 4.38 (q, *J* = 7.0 Hz, 2H, CH_2_), 7.08 (d, *J* = 6.0 Hz, 1H, H_6_), 7.13 (s, 2H, H_12_, H_16_), 7.20 (dd, *J* = 6.5; 4.5 Hz, 1H, H_20_), 7.46 (dd, *J* = 9.0; 7.0 Hz, 1H, H_7_), 7.64 (s, 1H, H_2_), 7.72 (d, *J* = 8.0 Hz, 1H, H_22_), 7.91 (t, *J* = 7.5 Hz, 1H, H_21_), 8.27 (d, *J* = 4.0 Hz, 1H, H_19_), 8.47 (d, *J* = 8.5 Hz, 1H, H_8_). ^13^C NMR (125 MHz, CDCl_3_): *δ* 14.7 CH_3_, 56.5 2 x OMe, 60.2 CH_2_, 61.1 OMe, 105.9 C_1_, 107.1 C_12_, C_16_, 118.1 C_6_, 120.2 C_8_, 122.2 C_22_, 124.0 C_20_, 126.5 C_7_, C_3_, 127.3 C_2_, 133.3 C_11_, 138.6 C_5_, 138.7 C_21_, 141.4 C_9_, 141.8 C_14_, 148.5 C_19_, 153.0 C_13_, C_15_, 155.2 C_17_, 164.4 COO, 183.4 C_10_. Anal. Calcd. for C_26_H_24_N_2_O_6_: C, 67.82; H, 5.25; N, 6.08. Found: C, 67.81; H, 5.23; N, 6.11.

*Ethyl 3-(3,5-dimethoxybenzoyl)-5-(pyridin-2-yl)indolizine-1-carboxylate (***14b***).* Yellow solid, yield 77%, mp 149–150 °C. IR *ν* (cm^−1^): 2974, 1715, 1630, 1595, 1421, 1188, 1155, 754. ^1^H NMR (500 MHz, CDCl_3_): *δ* 1.39 (t, *J* = 7.0 Hz, 3H, CH_3_), 3.81 (s, 6H, 2 × OMe), 4.37 (q, *J* = 7.0 Hz, 2H, CH_2_), 6.66 (bs, 1H, H_14_), 7.00 (d, *J* = 2.0 Hz, 2H, H_12_, H_16_), 7.09 (d, *J* = 7.0 Hz, 1H, H_6_), 7.18 (dd, *J* = 6.5; 5.5 Hz, 1H, H_20_), 7.47 (dd, *J* = 8.5; 7.5 Hz, 1H, H_7_), 7.65 (s, 1H, H_2_), 7.71 (d, *J* = 8.0 Hz, 1H, H_22_), 7.88 (t, *J* = 7.5 Hz, 1H, H_21_), 8.30 (d, *J* = 4.5 Hz, 1H, H_19_), 8.47 (d, *J* = 8.5 Hz, 1H, H_8_). ^13^C NMR (125 MHz, CDCl_3_): 14.7 CH_3_, 55.82 × OMe, 60.2 CH_2_, 105.1 C_14_, 105.9 C_1_, 107.3 C_12_, C_16_, 118.2 C_6_, 120.2 C_8_, 122.0 C_22_, 123.9 C_20_, 126.5 C_3_, 126.7 C_7_, 127.8 C_2_, 138.5 C_21_, 138.7 C_5_, 140.2 C_11_, 141.6 C_9_, 148.5 C_19_, 155.2 C_17_, 160.7 C_13_, C_15_, 164.4 COO, 183.6 C_10_. Anal. Calcd. for C_25_H_22_N_2_O_5_: C, 69.76; H, 5.15; N, 6.51. Found: C, 69.78; H, 5.13; N, 6.54.

*Ethyl 3-(3,4-dimethoxybenzoyl)-5-(pyridin-2-yl)indolizine-1-carboxylate (***14c***).* Yellow solid, yield 35%. IR *ν* (cm^−1^): 2933, 1719, 1676, 1624, 1593, 1514, 1417, 1269, 1224, 1024, 766. ^1^H NMR (500 MHz, CDCl_3_): *δ* 1.39 (t, *J* = 7.0 Hz, 3H, CH_3_), 3.98 (s, 3H, OMe), 4.00 (s, 3H, OMe), 4.38 (q, *J* = 7.0 Hz, 2H, CH_2_), 6.95 (d, *J* = 8.5 Hz, 1H, H_15_), 7.08 (d, *J* = 6.0 Hz, 1H, H_6_), 7.19 (dd, *J* = 6.5; 4.5 Hz, 1H, H_20_), 7.42 (bs, 1H, H_12_), 7.47 (dd, *J* = 8.5; 7.0 Hz, 1H, H_7_), 7.48 (d, *J* = 8.0 Hz, 1H, H_16_), 7.64 (s, 1H, H_2_), 7.71 (d, *J* = 8.0 Hz, 1H, H_22_), 7.90 (t, *J* = 7.5 Hz, 1H, H_21_), 8.28 (d, *J* = 4.0 Hz, 1H, H_19_), 8.47 (d, *J* = 8.5 Hz, 1H, H_8_). ^13^C NMR (125 MHz, CDCl_3_): *δ* 14.6 CH_3_, 56.2 OMe, 56.3 OMe, 60.2 CH_2_, 106.0 C_1_, 110.3 C_15_, 111.9 C_12_, 118.2 C_6_, 120.2 C_8_, 122.1 C_22_, 123.5 C_16_, 124.0 C_20_, 126.5 C_3_, 126.6 C_7_, 127.6 C_2_, 132.5 C_11_, 138.7 C_5_, 138.6 C_21_, 141.5 C_9_, 148.5 C_19_, 149.1 C_13_, 153.3 C_14_, 155.2 C_17_, 164.3 COO, 184.5 C_10_. Anal. Calcd. for C_25_H_22_N_2_O_5_: C, 69.76; H, 5.15; N, 6.51. Found: C, 69.79; H, 5.12; N, 6.55.

*Ethyl 3-(4-bromobenzoyl)-5-(pyridin-2-yl)indolizine-1-carboxylate (***14d***)*^30^. Yellow crystals, yield 40%, mp 229–231 °C. IR *ν* (cm^−1^): 2925, 1725, 1694, 1650, 1586, 1419, 1342, 1070, 750. ^1^H NMR (500 MHz, DMSO-d_6_): *δ* 1.31 (t, *J* = 7.0 Hz, 3H, CH_3_), 4.29 (q, *J* = 7.0 Hz, 2H, CH_2_), 7.24 (dd, *J* = 7.0; 4.5 Hz, 1H, H_20_), 7.35 (dd, *J* = 7.5; 1.0 Hz, 1H, H_6_), 7.42 (s, 1H, H_2_), 7.67–7.70 (overlapped signals, 3H, H_12_, H_16_, H_7_), 7.74 (d, *J* = 8.5 Hz, 2H, H_13_, H_15_), 7.81 (d, *J* = 8.0 Hz, 1H, H_22_), 7.93 (dt, *J* = 7.5; 1.5 Hz, 1H, H_21_), 8.13 (d, *J* = 4.0 Hz, 1H, H_19_), 8.38 (dd, *J* = 9.0; 1.0 Hz, 1H, H_8_). ^13^C NMR (125 MHz, DMSO-d_6_): *δ* 14.5 CH_3_, 59.8 CH_2_, 104.4 C_1_, 118.3 C_6_, 119.0 C_8_, 122.2 C_22_, 124.3 C_20_, 125.1 C_2_, 126.2 C_3_, 126.8 C_14_, 127.7 C_7_, 131.2 C_12_, C_16_, 131.8 C_13_, C_15_, 136.2 C_11_, 138.4 C_21_, 138.8 C_5_, 140.4 C_9_, 148.3 C_19_, 154.2 C_17_, 163.3 COO, 182.2 C_10_. Anal. Calcd. for C_23_H_17_BrN_2_O_3_: C, 61.48; H, 3.81; N, 6.23. Found: C, 61.51; H, 3.77; N, 6.25.

*4-(1-(Ethoxycarbonyl)-3-(3,4,5-trimethoxybenzoyl)indolizin-7-yl)-1-(2-oxo-2-(3,4,5-trimethoxy phenylethyl)pyridin-1-ium bromide (***15a***).* Orange solid, yield 70%, mp 188 °C. IR *ν* (cm^−1^): 2986, 2941, 1713, 1640, 1583, 1530, 1508, 1465, 1412, 1346, 1323, 1207, 1130. ^1^H NMR (500 MHz, CDCl_3_): *δ* 1.42 (t, *J* = 7.0 Hz, 3H, CH_3_), 3.92 (s, 6H, 2 × OMe), 3.93 (s, 3H, OMe), 3.96 (s, 6H, 2 × OMe), 3.97 (s, 3H, OMe), 4.42 (q, *J* = 7.0 Hz, 2H, CH_2_), 7.09 (s, 2H, H_12_, H_16_), 7.26 (s, 2H, H_23_), 7.47–7.49 (3H, overlapped signals, H_6_, H_26_, H_30_), 7.89 (s, 1H, H_2_), 8.35 (bs, 2H, H_18_, H_22_), 8.89 (bs, 1H, H_8_), 9.35 (bs, 2H, H_19_, H_21_,), 9.92 (d, *J* = 7.0 Hz, 1H, H_5_). ^13^C NMR (125 MHz, CDCl_3_): *δ* 14.7 CH_3_, 56.62 × OMe, 57.22 × OMe, 60.9 CH_2_, 61.1 OMe, 61.2 OMe, 66.4 C_23_, 106.9 C_26_, C_30_, 107.0 C_12_, C_16_, 109.8 C_1_, 112.4 C_6_, 119.9 C_8_, 123.9 C_3_, 124.1 C_18_, C_22_, 128.4 C_2_, 128.6 C_25_, 130.0 C_5_, 130.9 C_7_, 134.2 C_11_, 138.2 C_9_, 142.1 C_14_, 144.4 C_28_, 147.0 C_19_, C_21_, 153.3 C_13_, C_15_, 153.6 C_27_, C_29_, 153.9 C_17_, 163.5 COO, 185.0 C_10_, 189.3 C_24_. Anal. Calcd. for C_37_H_37_BrN_2_O_10_: C, 59.28; H, 4.98; N, 3.74. Found: C, 59.32; H, 4.87; N, 3.79.

*1-(2-(3,5-Dimethoxyphenyl)-2-oxoethyl)-4-(1-(ethoxycarbonyl)-3-(3,4,5-trimethoxybenzoyl) indolizin-7-yl)pyridin-1-ium bromide (***15b***).* Orange solid, yield 65%, mp 175 °C. IR *ν* (cm^−1^): 2928, 1700, 1638, 1529, 1457, 1351, 1207, 1016. ^1^H NMR (400 MHz, DMSO-d_6_): *δ* 1.37 (t, *J* = 6.8 Hz, 3H, CH_3_), 3.80 (s, 3H, OMe), 3.86 (s, 12H, 4 × OMe), 4.37 (q, *J* = 6.8 Hz, 2H, CH_2_), 6.50 (s, 2H, H_23_), 6.95 (s, 1H, H_28_), 7.15 (s, 2H, H_12_, H_16_), 7.21 (s, 2H, H_26_, H_30_), 7.84 (s, 1H, H_2_), 7.96 (bs, H_6_), 8.80 (bs, 2H, H_18_, H_22_), 8.95 (bs, 1H, H_8_), 9.11 (bs, 2H, H_19_, H_21_), 9.87 (bs, 1H, H_5_). ^13^C NMR (100 MHz, DMSO-d_6_): *δ* 14.3 CH_3_, 55.82 × OMe, 56.23 × OMe, 60.3 CH_2_, 65.9 C_23_, 106.1 C_26_, C_28_, C_30_, 106.7 C_12_, C_16_, 108.2 C_1_, 113.7 C_6_, 119.0 C_8_, 123.1 C_3_, 124.8 C_18_, C_22_, 127.9 C_2_, 129.2 C_5_, 132.0 C_7_, 133.9 C_11_, 135.4 C_25_, 137.8 C_9_, 141.0 C_14_, 146.6 C_19_, C_21_, 152.5 C_17_, 152.7 C_13_, C_15_, 160.9 C_27_, C_29_, 162.8 COO, 184.1 C_10_, 190.6 C_24_. Anal. Calcd. for C_36_H_35_BrN_2_O_9_: C, 60.09; H, 4.90; N, 3.89. Found: C, 60.12; H, 4.87; N, 3.94.

*1-(2-(3,4-Dimethoxyphenyl)-2-oxoethyl)-4-(1-(ethoxycarbonyl)-3-(3,4,5-trimethoxybenzoyl) indolizin-7-yl)pyridin-1-ium bromide (***15c***).* Orange solid, yield 50%, mp 236–239 °C. IR *ν* (cm^−1^): 2925, 1699, 1638, 1582, 1523, 1460, 1345, 1272, 1205. ^1^H NMR (400 MHz, DMSO-d_6_): *δ* 1.37 (t, *J* = 6.8 Hz, 3H, CH_3_), 3.80 (s, 3H, OMe), 3.86 (s, 9H, 3 × OMe), 3.92 (s, 3H, OMe), 4.37 (q, *J* = 6.8 Hz, 2H, CH_2_), 6.47 (s, 2H, H_23_), 7.16 (s, 2H, H_12_, H_16_), 7.24 (bs, 1H, H_29_), 7.55 (bs, 1H, H_26_), 7.81 (bs, 1H, H_30_), 7.85 (s, 1H, H_2_), 7.96 (bs, 1H, H_6_), 8.80 (bs, 2H, H_18_, H_22_), 8.97 (bs, 1H, H_8_), 9.11 (bs, 2H, H_19_, H_21_), 9.89 (bs, 1H, H_5_). ^13^C NMR (100 MHz, DMSO-d_6_): *δ* 14.3 CH_3_, 55.8 OMe, 56.0 2 × OMe, 56.22 × OMe, 60.2 CH_2_, 65.4 C_23_, 110.3 C_26_, 106.7 C_12_, C_16_, 108.2 C_1_, 111.3 C_29_, 113.7 C_6_, 118.9 C_8_, 123.2 C_3_, 123.4 C_30_, 124.7 C_18_, C_22_, 126.2 C_25_, 127.9 C_2_, 129.3 C_5_, 132.0 C_7_, 133.9 C_11_, 137.8 C_9_, 141.0 C_14_, 146.6 C_19_, C_21_, 148.9 C_27_, 152.4 C_28_, 152.7 C_13_, C_15_, C_17_, 162.8 COO, 184.1 C_10_, 189.0 C_24_. Anal. Calcd. for C_36_H_35_BrN_2_O_9_: C, 60.09; H, 4.90; N, 3.89. Found: C, 60.10; H, 4.85; N, 3.93.

*4-(3-(3,5-Dimethoxybenzoyl)-1-(ethoxycarbonyl)indolizin-7-yl)-1-(2-oxo-2-(3,4,5-trimethoxyphenyl)ethyl)pyridin-1-ium bromide (***15d***).* Orange solid, yield 84%, mp 176–178 °C. IR *ν* (cm^−1^): 2913, 1696, 1683, 1639, 1590, 1528, 1454, 1402, 1352, 1207, 1159, 1130. ^1^H NMR (500 MHz, DMSO-d_6_): *δ* 1.36 (t, *J* = 7.0 Hz, 3H, CH_3_), 3.81 (s, 3H, OMe), 3.84 (s, 6H, 2 × OMe), 3.92 (s, 6H, 2 × OMe), 4.37 (q, *J* = 7.0 Hz, 2H, CH_2_), 6.52 (s, 2H, H_23_), 6.83 (t, *J* = 2.0 Hz, 1H, H_14_), 6.93 (d, *J* = 2.0 Hz, 2H, H_12_, H_16_), 7.39 (s, 2H, H_26_, H_30_), 7.76 (s, 1H, H_2_), 7.97 (dd, *J* = 7.5; 2.0 Hz, H_6_), 8.81 (d, *J* = 7.0 Hz, 2H, H_18_, H_22_), 8.97 (bs, 1H, H_8_), 9.10 (d, *J* = 6.5 Hz, 2H, H_19_, H_21_), 9.92 (d, *J* = 7.5 Hz, 1H, H_5_). ^13^C NMR (125 MHz, DMSO-d_6_): *δ* 14.4 CH_3_, 55.6 2 × OMe, 56.4 2 × OMe, 60.3 CH_2_, 60.4 OMe, 65.7 C_23_, 103.5 C_14_, 106.0 C_26_, C_30_, 106.8 C_12_, C_16_, 108.2 C_1_, 113.9 C_6_, 119.0 C_8_, 123.1 C_3_, 124.8 C_18_, C_22_, 128.1 C_2_, 128.8 C_25_, 129.3 C_5_, 132.2 C_7_, 138.0 C_9_, 140.8 C_11_, 143.2 C_28_, 146.6 C_19_, C_21_, 152.4 C_17_, 153.1 C_27_, C_29_, 160.4 C_13_, C_15_, 162.8 COO, 184.6 C_10_, 189.7 C_24_. Anal. Calcd. for C_36_H_35_BrN_2_O_9_: C, 60.09; H, 4.90; N, 3.88. Found: C, 60.03; H, 4.81; N, 4.84.

*4-(3-(3,5-Dimethoxybenzoyl)-1-(ethoxycarbonyl)indolizin-7-yl)-1-(2-(3,5-dimethoxyphenyl)-2-oxoethyl)pyridin-1-ium bromide (***15e***).* Orange solid, yield 75%, mp 149–150 °C. IR *ν* (cm^−1^): 2940, 1709, 1640, 1603, 1465, 1425, 1348, 1207, 1159, 1062, 758. ^1^H NMR (500 MHz, DMSO-d_6_): *δ* 1.36 (t, *J* = 7.0 Hz, 3H, CH_3_), 3.84 (s, 6H, 2 × OMe), 3.87 (s, 6H, 2 × OMe), 4.37 (q, *J* = 7.0 Hz, 2H, CH_2_), 6.48 (s, 2H, H_23_), 6.83 (t, *J* = 2.0 Hz, 1H, H_14_), 6.93 (d, *J* = 2.0 Hz, 2H, H_12_, H_16_), 6.95 (t, *J* = 2.0 Hz, 1H, H_28_), 7.21 (d, *J* = 2.0 Hz, 2H, H_26_, H_30_), 7.75 (s, 1H, H_2_), 7.97 (dd, *J* = 7.5; 1.5 Hz, H_6_), 8.80 (d, *J* = 6.5 Hz, 2H, H_18_, H_22_), 8.96 (bs, 1H, H_8_), 9.10 (d, *J* = 6.5 Hz, 2H, H_19_, H_21_), 9.91 (d, *J* = 7.5 Hz, 1H, H_5_). ^13^C NMR (DMSO-d_6_, 125 MHz): *δ* 14.4 CH_3_, 55.62 × OMe, 55.82 × OMe, 60.2 CH_2_, 65.9 C_23_, 103.5 C_14_, 106.1 C_26_, C_30_, 106.2 C_28_, 106.8 C_12_, C_16_, 108.2 C_1_, 113.8 C_6_, 119.0 C_8_, 123.1 C_3_, 124.8 C_18_, C_22_, 128.1 C_2_, 129.3 C_5_, 132.2 C_7_, 135.4 C_25_, 138.0 C_9_, 140.8 C_11_, 146.6 C_19_, C_21_, 152.5 C_17_, 160.4 C_13_, C_15_, 160.9 C_27_, C_29_, 162.8 COO, 184.6 C_10_, 190.5 C_24_. Anal. Calcd. for C_35_H_33_BrN_2_O_8_: C, 60.96; H, 4.82; N, 4.06. Found: C, 60.93; H, 4.77; N, 4.13.

*4-(3-(3,5-Dimethoxybenzoyl)-1-(ethoxycarbonyl)indolizin-7-yl)-1-(2-(3,4-dimethoxyphenyl)-2-oxoethyl)pyridin-1-ium bromide (***15f***).* Orange solid, yield 60%, mp 220–223 °C. IR *ν* (cm^−1^): 2924, 2851, 1696, 1640, 1596, 1524, 1463, 1401, 1349, 1200, 1157. ^1^H NMR (500 MHz, DMSO-d_6_): *δ* 1.36 (t, *J* = 7.0 Hz, 3H, CH_3_), 3.84 (s, 6H, 2 × OMe), 3.86 (s, 3H, OMe), 3.92 (s, 3H, OMe), 4.37 (q, *J* = 7.0 Hz, 2H, CH_2_), 6.45 (s, 2H, H_23_), 6.83 (bs, 1H, H_14_), 6.93 (d, *J* = 2.0 Hz, 2H, H_12_, H_16_), 7.24 (d, *J* = 8.5 Hz, 1H, H_29_), 7.54 (bs, 1H, H_26_), 7.76 (s, 1H, H_2_), 7.79 (dd, *J* = 8.0; 1.0 Hz, 1H, H_30_), 7.97 (dd, *J* = 7.5; 1.5 Hz, H_6_), 8.79 (d, *J* = 6.5 Hz, 2H, H_18_, H_22_), 8.97 (bs, 1H, H_8_), 9.09 (d, *J* = 6.5 Hz, 2H, H_19_, H_21_), 9.92 (d, *J* = 7.5 Hz, 1H, H_5_). ^13^C NMR (125 MHz DMSO-d_6_): *δ* 14.4 CH_3_, 55.72 × OMe, 55.8 OMe, 56.1 OMe, 60.3 CH_2_, 65.6 C_23_, 103.6 C_14_, 106.9 C_12_, C_16_, 108.0 C_1_, 110.1 C_26_, 111.6 C_29_, 113.7 C_6_, 119.0 C_8_, 123.2 C_3_, 123.5 C_30_, 124.8 C_18_, C_22_, 126.3 C_25_, 128.2 C_2_, 129.3 C_5_, 132.3 C_7_, 138.0 C_9_, 140.8 C_11_, 146.7 C_19_, C_21_, 149.0 C_27_, 152.4 C_28_, 154.3 C_17_, 160.5 C_13_, C_15_, 162.9 COO, 184.9 C_10_, 189.1 C_24_. Anal. Calcd. for C_35_H_33_BrN_2_O_8_: C, 60.96; H, 4.82; N, 4.06; Found: C, 60.93; H, 4.80; N, 4.13.

*4-(3-(3,4-Dimethoxybenzoyl)-1-(ethoxycarbonyl)indolizin-7-yl)-1-(2-oxo-2-(3,4,5-trimethoxyphenyl)ethyl)pyridin-1-ium bromide (***15g***).* Orange solid, yield 70%, mp 132–137 °C. IR *ν* (cm^−1^): 2924, 1698, 1640, 1619, 1456, 1410, 1344, 1265, 1206, 1124. ^1^H NMR (500 MHz, CDCl_3_): *δ* 1.44 (t, *J* = 7.0 Hz, 3H, CH_3_), 3.93 (s, 3H, OMe), 3.97 (s, 6H, 2 × OMe), 4.00 (s, 6H, 2 × OMe), 4.42 (q, *J* = 7.0 Hz, 2H, CH_2_), 7.23 (s, 2H, H_23_), 6.95 (d, *J* = 8.5 Hz, 1H, H_15_), 7.42–7.47 (3H, overlapped signals, H_12_, H_16_, H_6_), 7.49 (bs, 2H, H_26_, H_30_), 7.84 (s, 1H, H_2_), 8.32 (d, *J* = 5.5 Hz, 2H, H_18_, H_22_), 8.84 (bs, 1H, H_8_), 9.32 (d, *J* = 5.0 Hz, 2H, H_19_, H_21_), 9.87 (d, *J* = 7.5 Hz, 1H, H_5_). ^13^C NMR (125 MHz, CDCl_3_): *δ* 14.7 CH_3_, 56.1 OMe, 56.22 × OMe, 56.92 × OMe, 60.8 CH_2_, 66.4 C_23_, 106.7 C_26_, C_30_, 109.3 C_1_, 110.1 C_15_, 111.7 C_12_, C_6_, 119.4 C_8_, 123.7 C_16_, C_3_, 123.8 C_18_, C_22_, 128.1 C_25_, 128.4 C_2_, 129.7 C_5_, 131.5 C_11_, 130.4 C_7_, 137.8 C_9_, 144.1 C_28_, 146.9 C_19_, C_21_, 149.3 C_13_, 152.9 C_1_, 153.3 C_17_, 153.4 C_27_, C_29_, 163.5 COO, 184.3 C_10_, 189.4 C_24_. Anal. Calcd. for C_36_H_35_BrN_2_O_9_: C, 60.09; H, 4.90; N, 3.89. Found: C, 60.13; H, 4.89; N, 3.93.

*4-(3-(3,4-Dimethoxybenzoyl)-1-(ethoxycarbonyl)indolizin-7-yl)-1-(2-(3,5-dimethoxyphenyl)-2-oxoethyl)pyridin-1-ium bromide (***15h***).* Orange solid, yield 62%, mp 138–139 °C. IR *ν* (cm^−1^): 2925, 1698, 1641, 1596, 1519, 1458, 1342, 1264, 1204, 1017. ^1^H NMR (500 MHz, CDCl_3_): *δ* 1.45 (t, *J* = 7.0 Hz, 3H, CH_3_), 3.86 (s, 6H, 2 × OMe), 3.98 (s, 3H, OMe), 4.00 (s, 3H, OMe), 4.42 (q, *J* = 7.0 Hz, 2H, CH_2_), 6.71 (s, 2H, H_23_), 6.63 (bs, 1H, H_28_), 6.94 (d, *J* = 8.5 Hz, 1H, H_15_), 7.28 (bs, 2H, H_26_, H_30_), 7.43–7.45 (3H, overlapped signals, H_12_, H_16_, H_6_), 7.81 (s, 1H, H_2_), 8.32 (bs, 2H, H_18_, H_22_), 8.82 (bs, 1H, H_8_), 9.35 (bs, 2H, H_19_, H_21_), 9.91 (d, *J* = 7.5 Hz, 1H, H_5_). ^13^C NMR (125 MHz, CDCl_3_): *δ* 14.7 CH_3_, 56.23 OMe, 56.24 2 x OMe, 56.3 OMe, 60.9 CH_2_, 66.8 C_23_, 106.6 C_26_, C_30_, 107.9 C_28_, 109.5 C_1_, 110.2 C_15_, 111.8 C_12_, 112.3 C_6_, 119.7 C_8_, 123.9 C_16_, C_3_, 124.0 C_18_, C_22_, 128.3 C_2_, 129.8 C_5_, 130.6 C_7_, 131.6 C_11_, 135.3 C_25_, 137.9 C_9_, 147.1 C_19_, C_21_, 149.4 C_13_, 153.0 C_14_, 153.6 C_17_, 161.3 C_27_, C_29_, 163.5 COO, 184.5 C_10_, 190.4 C_24_. Anal. Calcd. for C_35_H_33_BrN_2_O_8_: C, 60.96; H, 4.82; N, 4.06. Found: C, 60.95; H, 4.79; N, 4.09.

*4-(3-(3,4-Dimethoxybenzoyl)-1-(ethoxycarbonyl)indolizin-7-yl)-1-(2-(3,4-dimethoxyphenyl)-2-oxoethyl)pyridin-1-ium bromide (***15i***).* Orange solid, yield 80%, mp 247–250 °C. IR *ν* (cm^−1^): 2972, 1702, 1684, 1595, 1518, 1413, 1337, 1268, 1213, 1019. ^1^H NMR (500 MHz, DMSO-d_6_): *δ* 1.37 (t, *J* = 7.0 Hz, 3H, CH_3_), 3.85 (s, 3H, OMe), 3.87 (s, 3H, OMe), 3.90 (s, 3H, OMe), 3.92 (s, 3H, OMe), 4.38 (q, *J* = 7.0 Hz, 2H, CH_2_), 6.47 (s, 2H, H_23_), 7.18 (d, *J* = 8.5 Hz, 1H, H_15_), 7.24 (d, *J* = 8.5 Hz, 1H, H_29_), 7.43 (bs, 1H, H_12_), 7.52 (d, *J* = 8.5 Hz, 1H, H_16_), 7.54 (bs, 1H, H_26_), 7.79 (s, 1H, H_2_), 7.80 (d, *J* = 8.0 Hz, 1H, H_30_), 7.93 (*J* = 7.0 Hz, d, H_6_), 8.79 (d, *J* = 6.5 Hz, 2H, H_18_, H_22_), 8.96 (bs, 1H, H_8_), 9.10 (d, *J* = 6.5 Hz, 2H, H_19_, H_21_), 9.84 (d, *J* = 7.5 Hz, 1H, H_5_). ^13^C NMR (125 MHz, DMSO-d_6_): *δ* 14.4 CH_3_, 55.6 OMe, 55.7 OMe, 55.8 OMe, 56.0 OMe, 60.2 CH_2_, 65.4 C_23_, 107.9 C_1_, 110.3 C_26_, 111.0 C_15_, 111.3 C_29_, 111.7 C_12_, 113.4 C_6_, 119.0 C_8_, 123.4 C_3_, C_30_, 123.6 C_16_, 124.6 C_18_, C_22_, 126.3 C_25_, 127.3 C_2_, 129.2 C_5_, 131.0 C_11_, 131.8 C_7_, 137.7 C_9_, 146.6 C_19_, C_21_, 148.7 C_13_, 148.9 C_27_, 152.4 C_28_, 152.6 C_14_, 154.4 C_17_, 162.9 COO, 183.8 C_10_, 189.1 C_24_. Anal. Calcd. for C_35_H_33_BrN_2_O_8_: C, 60.96; H, 4.82; N, 4.06. Found: C, 60.90; H, 4.78; N, 4.11.

*4-(3-(4-Chlorobenzoyl)-1-(ethoxycarbonyl)indolizin-7-yl)-1-(2-(4-methoxy phenyl)-2-oxoethyl) pyridin-1-ium bromide (***15j***).* Yield 93%. All spectral data are in agreement with the literature^32^.

### Anticancer activity

The compounds were tested against a panel of 60 human cancer cell lines at the National Cancer Institute (NCI) (Rockville, MD). The cytotoxicity experiments were performed using a 48 h exposure protocol which consisted of a sulphorhodamine B assay^33–35^.

### Tubulin polymerisation assay

Microtubule assembly was studied using the tubulin polymerisation assay kit (Cytoskeleton Inc., Denver, CO, Cat. # BK006P), according to the manufacturer's instructions^36^^,^^37^.

**Figure 2.**
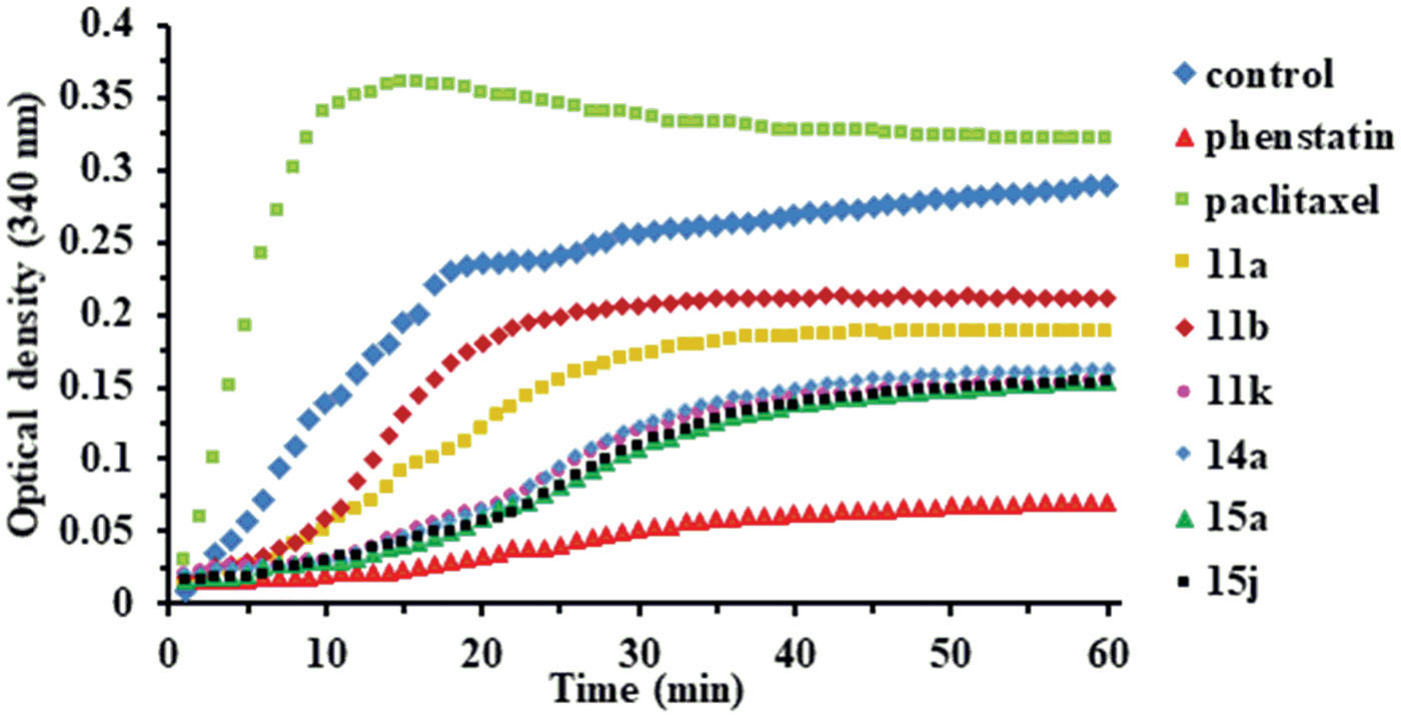
Effects of compounds **11a**, **11b**, **11k**, **14a**, **15a**, and **15j** (10^−5^ M) on microtubule dynamics using Paclitaxel (10^−5^ M) as microtubule stabilising agent and Phenstatin (10^−5^ M) as microtubule destabilising agent.

The polymerisation was monitored using FLUOstar Omega multi-mode microplate reader (BMG LABTECH, Ortenberg, Germany). The first step before the analysis is the pre-warming of the plate to 37 °C for 30 min. Plate temperature is essential for high polymerisation activity and reproducible results. The tubulin polymerisation buffer will be composed of general tubulin buffer, tubulin glycerol buffer, and GTP stock, at 4 °C. Another volume of 500 µl of general tubulin buffer is necessary for dilutions, at r.t. Ten microlitres of GTB will be pipetted into each well. Two of the wells will remain with GTB only, as controls. Into the rest, 10 µl of compounds of ×10 strength or Phenstatin, also of ×10 strength will be added. The final concentration of the compounds and Phenstatin will be 10 µM. The plate will be incubated at 37 °C for 2 min. Meanwhile, proceed to the dilution of 10 µl of the Paclitaxel Stock solution with 190 µl of r.t. general tubulin buffer, to be used in quantities of 10 µl of this per well. The tubulin will be defrosted until r.t., placed on ice and diluted with 420 TP cold buffer, reaching a final concentration of 3 mg/ml in 80 mM PIPES (piperazine-N,N′-bis(2-ethanesulfonic acid) sesquisodium salt), pH = 6.9, 2 mM MgCl_2_, 0.5 mM EGTA (ethylene glycol-bis (β-amino-ethyl ether) N,N,N′,N′-tetra-acetic acid, 1 mM GTP and 10.2% glycerol. The diluted tubulin will be used immediately by adding 120 µl into each of the wells with a multichannel pipette. The absorbance was measured at 340 nm for 1 h at 1 min intervals using a plate reader at 37 °C. Representative experiment (*n* = 3) is shown in Figure 2.

### Molecular modelling

Flexible-ligand docking experiments were performed as previously reported^24^, with slight modifications. Briefly, the 3D structures of the ligands were constructed in Avogadro v1.2.0^38^ and were energetically optimised in the MMFF94 force field until a local energy minimum was achieved. Autodock Vina^39^ was used for all docking experiments, using a 22 × 22 × 22 Å^3^ gridbox centred on the colchicine binding site of the α,β-tubulin heterodimer (PDB: 4O2B)^40^. The co-crystallised colchicine ligand and water molecules were removed during protein preparation for docking, and the target protein was kept rigid. Twenty poses were generated for each ligand, which were then ranked based on theoretical binding energy. The best ranked models were visually inspected in order to assess the consistency of the generated docking solutions relative to the docking poses of the known inhibitors colchicine and Phenstatin. In order to evaluate the quality of the docking protocol, colchicine was extracted from the crystal structure and re-docked into the binding site. RMSD between re-docked ligand and co-crystallised conformation was computed in PyMOL. Visual inspection, molecular graphics and analyses were made in the PyMOL Molecular Graphics System, Version 1.8.2 (Schrödinger, LLC, New York, NY) and Discovery Studio Visualiser Version 20.1.0.19295 (Dassault Systemes, BIOVIA Corp., San Diego, CA).

## Results and discussion

### Chemistry

**Scheme 1.**
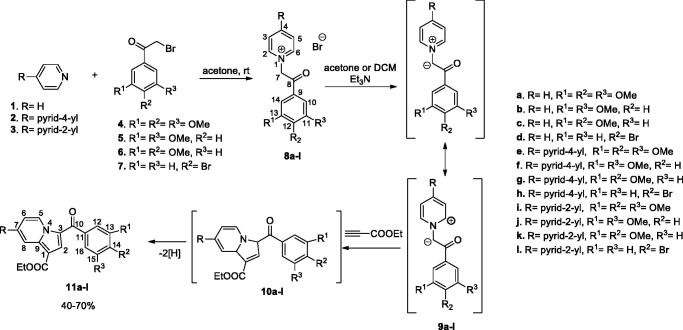
Synthesis pathway for indolizines **11a–l**.

**Scheme 2.**
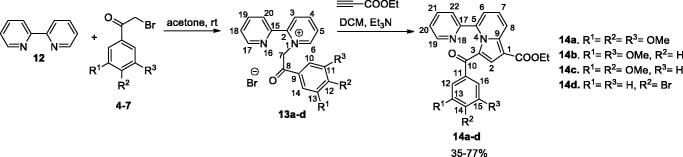
Synthesis of indolizines **14a–d**.

The pyridinium salts **8a–l** and **13a–d** were prepared through the direct reaction of pyridine **1**, 4,4′-bipyridine **2**, 2,4′-bipyridine **3**, or 2,2′-bipyridine **12**, respectively, with 2-bromo-acetophenones **4**–**7** in acetone, at r.t. (Schemes 1 and 2) (for spectral data of pyridinium salts **8a–l** and **13a–d** see Supplementary data). In the next step, for the synthesis of the indolizine ring, we used the 1,3-dipolar cycloaddition of the pyridinium ylides generated *in situ* in basic medium from the salts **8a–l** and **13a–d**, to ethyl propiolate (Schemes 1 and 2)^21^^,^^30^^,^^31^^,^^41^.

**Scheme 3.**
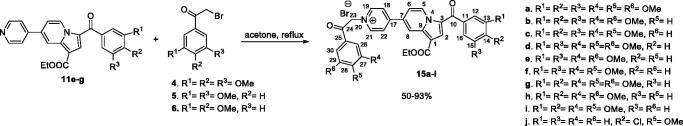
Synthesis of indolizines **15a–i**.

Indolizines **15a–i** were obtained in good yields using the substitution of halides **4–6** generated by the indolizines **11e–g** (Scheme 3)^32^^,^^42^. We also synthesised the previously reported compound **15j**^32^ using a similar procedure.

The structures of all new target compounds were fully confirmed by ^1^H and ^13^C NMR, IR and elemental analyses.

### Biological activity

#### Anticancer activity

All synthesised compounds were submitted to the NCI, and 13 compounds (**11a, b, d, e, f, i, j, k, l, 14a, 14d, 15a**, and **15j**) were selected for single dose (10^−5^ M) screening against a panel of 60 human tumour cell lines, representing leukaemia, melanoma and cancer of lung, colon, central nervous system, ovary, kidney, prostate, and breast^33^. Representative results for 11 of the compounds are summarised in Table 1.

**Table 1.** Results of the *in vitro* growth inhibition (GI %) of tested compounds against human cancer cell lines in the single-dose assay^a^.

Cell type	Cell line	GI (%) (10^–5^ M)^a^										
	Compound	11a	11b	11d	11e	11f	11i	11j	11k	14a	15a	15j
Leukaemia	CCRF-CEM	83	65	24	10	20	0	0	0	0	**100^b,p^**	**100^b,i′^**
	K-562	89	86	23	1	11	0	0	0	2	**100^b,q^**	**100^b,j′^**
	SR	76	84	32	30	30	0	0	14	5	**100^b,r^**	**100^b,k′^**
	HL-60(TB)	**100**^b,c^	**100**^b,l^	30	35	28	0	0	0	2	67	75
	MOLT-4	76	69	34	0	0	0	0	0	3	**100^b,s^**	89
	RPMI-8226	77	72	10	43	13	0	0	27	15	**100^b,t^**	77
Non-small cell lung cancer	A549/ATCC	74	64	11	20	11	0	14	53	0	68	68
	HOP-62	72	70	10	10	11	13	4	47	4	5	12
	NCI-H460	86	85	0	3	0	0	12	82	0	**100^b,u^**	**96**
	NCI-H522	74	59	25	21	19	34	7	**100^b,o^**	13	**100^b,v^**	29
Colon cancer	COLO205	**100**^b,d^	86	0	0	0	0	0	35	0	56	57
	HCT-116	81	88	19	24	3	11	0	0	3	**100^b,w^**	**95**
	HCT-15	71	74	17	1	0	0	0	17	3	17	25
	HT-29	**99**	**90**	23	8	12	0	0	23	0	**100^b,x^**	**100^b,l′^**
	SW-620	70	78	7	0	0	0	0	41	4	**100^b,z^**	**100^b,m′^**
	KM12	75	76	2	0	2	0	0	23	0	55	36
CNS cancer	SF-295	79	73	3	0	0	9	0	31	0	6	0
	SF-539	**100**^b,e^	55	3	6	0	12	11	70	0	70	10
	SNB-75	67	79	2	11	9	17	15	76	38	24	7
	U251	78	61	10	7	5	26	14	78	0	**100^b,a′^**	87
	SF-268	44	53	11	4	0	3	7	75	11	**100^b,b′^**	21
Melanoma	LOX IMVI	53	61	8	1	3	0	0	15	6	**100^b,c′^**	87
	M14	**100**^b,f^	96	0	0	0	5	0	0	0	45	43
	MDA-MB-435	**100**^b,g^	**100**^b,m^	0	0	0	0	0	29	11	31	24
	UACC-62	50	69	11	1	2	8	1	16	6	13	0
	SK-MEL-5	62	73	7	17	3	0	7	63	4	18	2
Ovarian cancer	OVCAR-3	**100**^b,h^	**100**^b,n^	0	0	0	0	0	21	0	18	63
	NCI/ADR-RES	89	88	8	1	0	0	0	34	5	6	11
	SK-OV-3	78	64	18	13	19	6	0	**94**	0	0	9
	OVCAR-8	67	58	12	9	6	0	9	63	8	93	23
	OVCAR-4	32	28	8	4	0	–	–	–	0	**100^b,d′^**	70
Renal cancer	A498	**100**^b,i^	71	2	14	0	0	6	58	0	20	26
	RXF393	**100**^b,j^	50	3	5	0	9	6	36	0	34	27
	ACHN	41	51	0	0	0	19	10	70	8	9	4
	786-0	65	64	0	10	0	9	0	0	–	**100^b,e′^**	**91**
	TK10	51	44	0	10	0	0	0	45	0	87	0
Breast cancer	MCF7	76	78	15	0	0	5	10	17	6	**100^b,f′^**	**99**
	MDA-MB-468	**100**^b,k^	70	3	30	9	10	6	25	0	57	29
	T-47D	53	51	16	16	0	9	20	77	0	50	17
	MDA-MB-231/ATCC	62	44	18	30	11	10	6	42	12	**100^b,g′^**	29
	BT-549	60	64	0	9	0	13	0	4	0	**100^b,h′^**	18
Prostate cancer	PC-3	88	60	17	15	12	2	9	13	2	46	49
	DU-145	75	46	0	0	0	0	0	35	0	55	70

^a^Data obtained from NCI’s *in vitro* 60 cell one dose screening at 10^−5^ M concentration; compounds **11l** and **14d** were also tested, but no GI was exhibited on the tested cell lines (results are not shown).

^b^Cytotoxic effect; cell growth percent: ^c^-25; ^d^-32; ^e^-7; ^f^ -22; ^g^-42; ^h^-6; ^i^-1; ^j^-0.4; ^k^-7; ^l^-14; ^m^-5; ^n^-5; ^o^-4; ^p^-15; ^q^-45; ^r^-16; ^s^-9; ^t^-22; ^u^-10; ^v^-21; ^w^-61; ^x^-10; ^z^-65; ^a′^-74, ^b′^-14, ^c′^-44; ^d′^-33; ^e′^-82; ^f′^-29; ^g′^-23; ^h′^-6; ^i′^-3; ^j′^-53; ^k′^-27; ^l′^-10; ^m′^-30.

The best values in terms of growth inhibition are highlighted in bold.

Indolizines **11a, b** showed a very good inhibition effect on almost all 60 lines, the best results being registered on leukaemia HL-60 (TB) cells, colon cancer COLO 205 cells, SNC cancer SF-539 cells, melanoma M14 and MDA-MB-435 cells, ovarian cancer cell OVCAR-3, renal cancer A498 and RXF393 and breast cancer MDA-MB-468 cells. Compound **11a** also exhibited a cytotoxic effect on all these lines, the best one being registered on melanoma MDA-MB-435 cells.

Interestingly, the substitution of indolizine heterocycle at position 7 with a pyrid-4-yl or pyrid-2-yl ring resulted in the loss of the activity, compounds **11e–f** and **11i**, **11j**, and **11l** (data not shown for compound **11l**) presenting almost no inhibition effect on the tested cell lines. As an exception, compound **11k** selectively inhibited the growth of NCI-H522 and NCI-H460 non-small cell lung cancer, SK-OV-3 ovarian cancer cells and T-47D breast cancer cells.

Substitution of the indolizine heterocycle at position 5 with a pyrid-2-yl ring also led to a loss of growth inhibition effect. Thus, compounds **14a** (Table 1) and **14d** showed no inhibition on tested cancer cells (data not shown for compound **14d**).

The presence of two 3,4,5-trimethoxybenzoyl groups in compound **15a** led to the best growth inhibition effect on the tested cancer cells. Compound **15a** is also distinguished by the high cytotoxic activity displayed on 18 cell lines, including cell lines from each panel except prostate cancer. Of the same serious, compound **15j** showed similar behaviour to **15a** on most line cells, but with much lower GI % values on NCI-H522 lung cancer cells, SF-268 CNS cancer cells, MDA-MB-231/ATCC and BT-549 breast cancer cells.

As shown in Table 1, the substitution of the 3,4,5-trimethoxyphenyl ring produced different effects in series of compounds C and D (Figure 1). Thus, in series C (**11a–d**), replacing the 3,4,5-trimethoxyphenyl ring with 3,5-dimethoxyphenyl does not alter the potency substantially, while substitution with 4-bromophenyl ring causes a dramatical loss of inhibitory properties. In series D (**11i–l**), the 3,4-dimethoxyphenyl ring appears to be the only one to confer selective inhibitory properties against the above mentioned cancer cell lines.

Showing the most significant growth inhibition, compounds **11a**, **15a**, and **15j** were selected for evaluation against 60 cell lines at five concentrations^33–35^. Results from the NCI-60 5-dose screen are shown in Table 2.

**Table 2.** Results of the 5-dose *in vitro* human cancer cell growth inhibition^a^ for compounds **11a**, **15a**, and **15j** and positive control Phenstatin.

Cell type	Compound	11a	11a	15a	15a	15j	15j	Phenstatin	Phenstatin
	Cell line	GI_50_ (μM)	LC_50_ (μM)	GI_50_ (μM)	LC_50_ (μM)	GI_50_ (μM)	LC_50_ (μM)	GI_50_ (μM)	LC_50_ (μM)
Leukaemia	K-562	0.036	>100	n.d.	n.d.	n.d.	n.d.	**<0.010**	>100
	HL-60(TB)	**0.032**	>100	2.58	>100	2.05	>100	**0.011**	>100
	SR	**0.023**	>100	2.90	>100	**0.33**	>100	**<0.010**	>100
	CCRF-CEM	0.055	>100	3.28	>100	**1.75**	>100	0.034	>100
	MOLT-4	0.077	>100	2.58	>100	2.04	>100	0.040	>100
	RPMI-8226	0.044	>100	n.d.	n.d.	n.d.	n.d.	0.037	>100
Non-small cell lung cancer	NCI-H460	0.042	>100	2.00	7.16	**1.63**	n.d.	0.033	>100
	NCI-H522	0.041	>100	2.13	>100	1.93	7.81	0.027	>100
	A549/ATCC	0.074	>100	3.29	>100	1.88	n.d.	0.057	>100
	HOP-62	0.051	>100	1.83	n.d.	1.78	n.d.	0.073	>100
Colon cancer	COLO205	**0.035**	>100	n.d.	n.d.	n.d.	n.d.	3.05	>100
	HCT-15	**0.035**	>100	2.62	>100	**1.64**	7.87	**<0.010**	>100
	HT29	0.037	>100	1.83	7.38	**1.63**	n.d.	2.95	>100
	SW-620	0.036	>100	1.76	**6.83**	**1.38**	7.70	**<0.010**	>100
	KM12	0.038	>100	1.81	**6.46**	1.93	n.d.	**<0.010**	>100
	HCT-116	0.053	>100	**1.72**	7.10	**1.61**	n.d.	0.038	>100
CNS cancer	SF-295	**0.032**	>100	1.86	n.d.	**1.70**	**6.46**	0.367	>100
	SF-539	0.053	>100	**1.61**	**6.77**	**1.68**	**6.35**	**0.011**	>100
	SNB-75	0.039	>100	**1.46**	7.77	**1.47**	**6.50**	**<0.010**	>100
	U251	0.053	>100	2.17	n.d.	**1.70**	7.39	0.043	>100
	SF268	0.088	>100	1.90	n.d.	1.86	n.d.	0.053	>100
	SNB-19	0.098	>100	2.12	n.d.	1.83	n.d.	0.031	>100
Melanoma	LOX IMVI	0.133	>100	1.87	n.d.	1.82	n.d.	**0.013**	>100
	M14	0.038	>100	1.91	n.d.	2.02	n.d.	**<0.010**	>100
	MDA-MB-435	**<0.010**	**20.4**	1.90	7.82	1.86	7.22	**<0.010**	>100
	UACC-62	**0.031**	>100	2.32	39.9	1.82	**6.79**	0.448	>100
	MALME-3M	0.089	>100	**1.73**	7.82	2.01	7.88	n.d.	>100
	SK-MEL-2	0.067	>100	2.60	>100	2.04	9.32	0.520	>100
	SK-MEL-5	0.041	>100	1.81	**6.93**	1.77	**6.60**	0.040	>100
Ovarian cancer	OVCAR-3	**0.033**	>100	1.83	7.11	1.88	7.08	0.021	>100
	NCI/ADR-RES	0.039	>100	>100	>100	3.16	>100	**0.012**	>100
	SK-OV-3	0.060	>100	19.6	>100	2.01	**6.51**	0.623	>100
Renal cancer	786-0	0.047	>100	**1.75**	**6.58**	1.80	n.d.	0.905	>100
	A498	**0.027**	>100	1.96	7.55	1.60	**6.29**	2.28	>100
	CAKI-1	0.066	>100	n.d.	n.d.	n.d.	n.d.	0.296	>100
	RXF 393	0.070	>100	1.66	7.27	**1.40**	**6.57**	**0.016**	>100
Breast cancer	MCF7	0.044	>100	1.79	n.d.	**1.40**	8.11	0.033	>100
	HS 578T	0.046	>100	**1.74**	25.0	1.81	>100	0.031	>100
	BT-549	0.060	>100	1.98	>100	1.87	n.d.	0.034	>100
	T-47D	0.051	>100	1.94	>100	1.83	n.d.	30.4	>100
	MDA-MB-468	0.094	>100	1.66	7.49	1.84	7.62	2.71	>100
Prostate cancer	PC-3	0.038	>100	2.61	>100	1.93	n.d.	0.045	>100
	DU-145	0.090	>100	1.80	**6.40**	**1.69**	**6.05**	0.039	>100

GI_50_: the molar concentration of tested compound causing 50% growth inhibition of tumour cells; LC_50_: the molar concentration of tested compound causing 50% death of tumour cells; n.d.: not determined.

The most significant values are highlighted in bold.

^a^ Data obtained from NCI’s *in vitro* 60 cell 5-dose screening^35–37^.

All three compounds displayed good antiproliferative properties. The best candidate in terms of growth inhibition properties was compound **11a**, with GI_50_ values < 100 nM against 47 cell lines, most notably on melanoma MDA-MB-435 (GI_50_<10 nM) and UACC-62 (GI_50_=31 nM) cells, leukaemia SR cells (GI_50_=23 nM), and renal cancer A498 cells (GI_50_=27 nM). Compound **11a** displayed selective cytotoxic activity on the melanoma MDA-MB-435 cell line (LC_50_=20.4 µM). Even if the overall antiproliferative activity of compound **11a** is lower in comparison with control Phenstatin, there are 11 cancer cell lines against which compound **11a** showed better GI_50_ values and several other lines against which compound **11a** had comparable GI_50_ values.

Although displaying excellent growth inhibitory effects at the 10^−5^ M single dose evaluation (Table 1), compounds **15a** and **15j** did not exhibit submicromolar GI_50_ values, except compound **15j** with GI_50_=0.33 µM against leukaemia SR cell line. Notably, both compounds showed considerable cytotoxic activity against all colon cancer, CNS cancer, renal cancer, and melanoma cell lines, but also on some cell lines from non-small cell lung cancer, ovarian cancer, breast cancer, and prostate cancer, respectively. This different behaviour of compounds **15a** and **15j** in comparison with compounds **11a** and Phenstatin, suggests different mechanisms of action.

#### *In vitro* tubulin polymerisation inhibition activity

In order to confirm if the observed anticancer activity of the above mentioned compounds is conferred by a microtubule-targeting mechanism, we evaluated the effect of the active compounds **11a**, **11b**, **11k**, **15a**, and **15j**, but also of the inactive compound **14a** (for comparison) on the assembly of tubulin. To confirm their influence on microtubule dynamics, two positive controls were used: paclitaxel (a tubulin stabiliser) and Phenstatin (a tubulin polymerisation inhibitor). As presented in Figure 2, Paclitaxel was found to stimulate tubulin polymerisation, while Phenstatin and all six tested compounds appeared to inhibit tubulin polymerisation.

Four compounds, namely **11k**, **14a**, **15a**, and **15j** showed a similar strong inhibitory behaviour on tubulin polymerisation, superior to the one obtained for compounds **11a** and **11b**. The obtained data clearly indicated that all tested target compounds effectively inhibit tubulin polymerisation *in vitro*.

### Molecular modelling

Molecular docking experiments revealed similar docking conformations of the tested compounds to previously reported anticancer pyrrolo[1,2-b]pyridazines (compounds type B, Figure 1), which are also thought to achieve anticancer activity by binding to the colchicine binding site of tubulin^24^. The used docking protocol was validated by computing the RMSD between re-docked colchicine and its co-crystallised conformation, which was 0.16 Å in our case. Generally, an RMSD value below 2 Å (the average resolution of a crystal structure) is considered acceptable^43^.

Compound **11a** is stabilised in the colchicine binding site of tubulin through H-bonding between its carbonyl moiety and βAsn258 and through extensive hydrophobic contacts with the accommodating pocket formed by βCys241, βLeu248, βLeu255, βAla250, βAla316, βIle318, βAla354, and βIle378, similar to Phenstatin. Moreover, the indolizine heterocycle extends towards the α subunit with the help of two polar interaction partners: βLys254 and αAsn101, and is further stabilised by the nonpolar chain of βLys352, which is engaged in an H-bond with αThr179 through its ε-NH_3_^+^ group. The 4-methoxy substituent does not engage in an H-bond with βCys241, as seen in the case of Phenstatin, but the binding orientation of this compound suggests that this bond could form if the binding site would be optimised. Further molecular dynamics simulations could be performed in order to investigate the formation and stability of this H-bond. Removal of the 4-methoxy group leads to a slight shift of the methoxy-substituted ring compared to **11a**, possibly to optimise hydrophobic contacts, as reflected by the similar theoretical binding energy (Table 3), but the formed H-bonds with βAsn258, βLys254, and αAsn101 are enough to maintain a conformation roughly overlapping with **11a**, which could account for the observed biological activity exhibited by this compound. The bromo-substituted compound **11d** was accommodated more deeply in the colchicine binding pocket, exclusively through hydrophobic interactions, having one of the lowest theoretical binding energies of all docked compounds. The absence of polar contacts upon accommodation at the tubulin binding site could explain the low biological properties exhibited by this compound.

**Table 3.** Binding orientation, energy, and amino acid contacts for tested compounds, as predicted by molecular docking experiments.





For binding orientation, the α,β-tubulin heterodimer is shown as ribbons; aminoacids and ligands are represented as sticks; for 2D interaction diagrams, colours are as follows: conventional hydrogen bonds – green, carbon–hydrogen bonds – pale green; hydrophobic interactions – light pink; amide–π stacking – dark pink; anion–π stacking: orange; π–sulphur stacking: dark yellow.

Docking experiments for compounds **11e**, **11f**, **11i**, **11j**, and **11k** did not reveal any conformations in which the methoxy-substituted cycle would overlap with the one in the colchicine binding site as to permit a polar interaction with βCys241. Instead, this aromatic moiety was oriented towards α subunit of the binding pocket, being stabilised by H-bonds with βLys254 (**11e**, **11i**) or αAsn101 (**11j**), as well as weak hydrogen bonding interactions with αSer178, βGln247 and the nonexchangeable GTP molecule. The indolizine moiety is stabilised by extensive hydrophobic contacts and, in the case of **11f, 11k**, by additional amide stacking with the backbone of βLys254. The pyridyl ring is positioned deep in the colchicine binding site, away from the α/β interface, and is stabilised through hydrophobic interactions with residues in the β subunit. Since all these compounds have good binding energies, yet lack activity, it could be postulated that a polar interaction with βCys241, or at least the positioning of possible polar interaction partners in the proximity of this residue is crucial for the observed anticancer activity, as has been seen for other colchicine binding site inhibitors^44–46^. However, an exception can be seen at compound **11k**, which showed selective activity against nine cancer cell lines (GI > 70%), and also inhibited tubulin polymerisation *in vitro*, but did not form a favourable contact with this residue in our docking experiments. Further mutagenesis experiments could be performed in order to describe the impact of this residue on the binding properties of the tested compounds. Despite its potent anticancer activity, the low theoretical binding score of **11a** compared to **11e–k** suggests that its cytotoxicity may involve other cellular targets or pathways other than the αβ-tubulin heterodimer. At the same time, since cancer cells preferentially express different β-tubulin isoforms, it would be possible that this compound binds with greater affinity to other isoforms. This aspect could be further studied *in silico*, as has been done for DAMA-colchicine^47^ and other colchicine binding site microtubule depolymerising agents^48^.

Compound **14a** was accommodated in a similar fashion to compounds **11a,b,d**, preserving the interactions of the methoxy-substituted moiety with the hydrophobic pocket formed by βCys241, βLeu248, βLeu255, βAla250, βAla316, βIle318, βAla354, and βIle378, but its indolizine ring rotated as to permit the interaction between the pyrid-2-yl ring and βMet259 through pi-sulphur stacking. This rotation also led to the formation of an H-bond between the carboxylate moiety of this compound and βGln247. While the *in vitro* tubulin polymerisation assay results are in agreement with the docking observations, the lack of anticancer activity in the case of compound **14a** remains to be elucidated.

Compound **15a** occupied the colchicine binding site similar to compounds **11a,b,d** and Phenstatin, engaging in H-bonds with βCys241 and βAsn258, and forming hydrophobic contacts with βLeu248, βLeu255, βAla250, βAla316, βIle318, and βAla354. The additional 3,4,5-trimethoxybenzoyl group reached towards the H10 helix of the β-tubulin subunit to form H-bonds with βThr353 and βGln336, as well as pi-anion stacking with the sidechain of βAsp329. The indolizine moiety was stabilised by amide-pi stacking with the backbone of αThr179. This compound had the one of the lowest binding energies of all tested molecules (–10.2 kcal/mol), being surpassed only by compound **15j** (–10.7 kcal/mol). Interestingly, compound **15j** forms a hydrophobic interaction with βCys241 and maintains many of the polar and hydrophobic contacts observed at **15a**, being accommodated in the same extended binding site-spanning conformation. Additional molecular dynamics experiments should be performed in order to confirm the stability of the observed interactions, especially with βCys241.

## Conclusions

Twenty-six new substituted Phenstatin analogues with an indolizine core were synthesised and submitted to NCI for anticancer activity evaluation. Thirteen compounds were selected and tested against a panel of 60 human cancer cells. Tubulin polymerisation assays and docking studies were also performed for the active compounds. Compounds **11a**, **11b**, **15a**, and **15j** showed excellent inhibitory properties on a broad range of cancer cell lines, and tubulin polymerisation assays revealed significant inhibitory effects on tubulin assembly for these compounds. This mechanism of action is further supported by docking experiments, which showed that all four compounds fit well to the colchicine binding site of tubulin. Interestingly, substitution of the indolizine heterocycle at position 7 with a pyrid-4-yl or pyrid-2-yl, or at position 5 with a pyrid-2-yl ring resulted in the loss of anticancer activity. As an exception, compound **11k** showed a good inhibitory profile on tubulin polymerisation, but only selectively inhibited the growth of NCI-H522 and NCI-H460 non-small cell lung cancer, SK-OV-3 ovarian cancer cells, and T-47D breast cancer cell lines. Interestingly, inhibitory tubulin polymerisation properties, as well as a good compatibility for the colchicine binding pocket of tubulin are shown by **14a**, but this compound is basically inactive against the tested cancer cells. Taken together, these results offer new SAR insights into this class of compounds and prove that using a strategy of structural combination can generate new colchicine site tubulin polymerisation inhibitors, as well as highly cytotoxic molecules against various cancer cells, which could aid the general research community in their ongoing anticancer efforts.

## Supplementary Material

Supplemental Material
