# Supplementary material for: Cytotoxic substituted indolizines as new colchicine site tubulin polymerisation inhibitors
Source: J Enzyme Inhib Med Chem. 2020 Aug 4;35(1):1581–95. doi: 10.1080/14756366.2020.1801671 (PMC7470029; doi:10.1080/14756366.2020.1801671)

SUPPLEMENTARY MATERIAL

Cytotoxic substituted indolizines as new colchicine site tubulin polymerization inhibitors

Monica-Cornelia Sardaru[a,b], A. -M. Craciun[a,b], C. -M. Al Matarneh[a,b], I. A. Sandu[b], R. –M. Amarandi[a,c], L. Popovici[a], C. I. Ciobanu[d], D. Peptanariu[b], M. Pinteala[b], I. I. Mangalagiu[a], R. Danac[a*]

[a]*Department of Chemistry, Faculty of Chemistry, "Al. I. Cuza" University of Iasi, 11 Carol I, Iasi 700506, Romania;* [b]*"Petru Poni" Institute of Macromolecular Chemistry of Romanian Academy, 41A Grigore Ghica Voda Alley, Iasi 700487, Romania;* [c]*TRANSCEND Research Center, Regional Institute of Oncology, 2-4 General Henri Mathias Berthelot Street, Iași 700483, Romania;* [d]*Research Department, Faculty of Chemistry, "Al. I. Cuza" University of Iasi, 11 Carol I, Iasi 700506, Romania*

*E-mail: rdanac@uaic.ro; Tel: +40232201342

Spectral data for compounds **8a-l** and **13a-d**	**S2**
^{1}H NMR and ^{13}C NMR spectra of compound **11a**	**S8**
^{1}H NMR and ^{13}C NMR spectra of compound **11b**	**S9**
^{1}H NMR and ^{13}C NMR spectra of compound **11k**	**S10**
^{1}H NMR and ^{13}C NMR spectra of compound **14a**	**S11**
^{1}H NMR and ^{13}C NMR spectra of compound **15a**	**S12**
^{1}H NMR and ^{13}C NMR spectra of compound **15j**	**S13**

Spectral data for compounds 8a-l and 13a-d

1-(2-oxo-2-(3,4,5-trimethoxyphenyl)ethyl)pyridin-1-ium bromide (8a). Yield 65%. All spectral data are in agreement with the literature [1].

1-(2-oxo-2-(3,5-dimethoxyphenyl)ethyl)pyridin-1-ium bromide (8b). Cream solid, yield 60%, mp 240-244 ºC. IR ν(cm-1): 1688, 1632, 1593, 1493, 1356, 1296, 1182, 1153, 1024. ^{1}H NMR (500 MHz, DMSO-d6): δ 3.85 (s, 6H, 2 x OMe), 6.50 (s, 2H, H_7), 6.93 (s, 1H, H_{12}), 7.18 (ad, J = 1.5 Hz, 2H, H_{10}, H_{14}), 8.27 (t, J = 7.5 Hz, 2H, H_3, H_5), 8.96 (t, J = 7.0 Hz, 1H, H_4), 9.00 (d, J = 6.0 Hz, 2H, H2, H_6). ^{13}C NMR (125 MHz, DMSO-d6): δ 55.8 2 x OMe, 66.4 C_7, 106.1 C_{10}, C_{14}, 106.2 C_{12}, 127.9 C_3, C_5, 135.4 C_9, 146.3 C_2, C_6, 146.5 C_4, 160.9 C_{11}, C_{13}, 190.5 C_8. Anal. Calcd. for $C_{15}H_{16}BrNO_3$: C, 53.27; H, 4.77; N, 4.14. Found: C, 53.25; H, 4.78; N, 4.18.

1-(2-(3,4-dimethoxyphenyl)-2-oxoethyl)pyridin-1-ium bromide (8c). Yellow solid, yield 30%, mp 194-196 ºC. IR ν(cm-1): 3038, 2953, 1591, 1580, 1524, 1493, 1348, 1279, 1180, 1138, 1013. ^{1}H NMR (500 MHz, DMSO-d6): δ 3.85 (3H, s, OMe), 3.90 (3H, s, OMe), 6.49 (s, 2H, H_7), 7.23 (d, J = 8.5 Hz, 1H, H_{13}), 7.52 (s, 1H, H_{10}), 7.77 (d, J = 8.5 Hz, 1H, H_{14}), 8.28 (t, J = 6.5 Hz, 2H, H_3, H_5). 8.73 (t, J = 7.5 Hz, 1H, H_4), 9.02 (d, J = 5.5 Hz, 2H, H_2, H_6). ^{13}C NMR (125 MHz, DMSO-d6): δ 55.7 OMe, 56.0 OMe, 65.9 C_7, 110.2 C_{10}, 111.2 C_{13}, 123.3 C_{14}, 126.2 C_9, 127.8 C_3, C_5, 148.8 C_{11}, 146.2 C_2, C_6, 146.3 C_4, 154.3 C_{12}, 189.0 C_8. Anal. Calcd. for $C_{15}H_{16}BrNO_3$: C, 53.27; H, 4.77; N, 4.14. Found: C, 53.25; H, 4.78; N, 4.18.

1-(2-(4-bromophenyl)-2-oxoethyl)pyridin-1-ium bromide (8d). White solid, yield 60%, mp 240-243 ºC. IR ν(cm-1): 3020, 2905, 1692, 1636, 1584, 1489, 1234, 1179, 1070. ^{1}H NMR (400 MHz, DMSO-d6): δ 6.46 (s, 2H, H_7), 7.90 (d, J = 8.8 Hz, 2H, H_{11}, H_{13}), 7.99 (d, J = 8.8 Hz, 2H, H_{10}, H_{14}), 8.27 (dd, J = 7.6; 6.8 Hz, 2H, H_3, H_5), 8.74 (t, J = 8.0 Hz, 1H, H_4), 8.98 (d, J = 5.6 Hz, 2H, H_2, H_6). ^{13}C NMR (100 MHz, DMSO-d6): δ 66.1 C_7, 127.8 C_3, C_5, 128.8 C_{12}, 130.2 C_{10}, C_{14}, 132.2 C_{11}, C_{13}, 132.6 C_9, 146.2 C_2, C_6, 146.4 C_4, 190.1 C_8. Anal. Calcd. for $C_{13}H_{11}Br_2NO$: C, 43.73; H, 3.11; N, 3.92. Found: C, 43.70; H, 3.08; N, 3.91.

1-(2-oxo-2-(3,4,5-trimethoxyphenyl)ethyl)-[4,4'-bipyridin]-1-ium bromide (8e). Cream solid, yield 50%, mp 240-242 ºC; IR ν(cm-1): 3010, 2978, 2940, 1688, 1641, 1584, 1462, 1415, 1344, 1317, 1161, 1119; 1H NMR (400 MHz, DMSO-d6) δppm: 3.81 (s, 3H, OMe), 3.91 (s, 6H, 2 x OMe), 6.58 (s, 2H, H7), 7.39 (s, 2H, H10, H14), 8.18 (bs, 2H, 2 x Hpy), 8.78 (bs, 2H, H3, H5), 8.97 (bs, 2H, 2 x Hpy), 9.36 (bs, 2H, H2, H6); 13C NMR (100 MHz, DMSO-d6) δppm: 56.4 (2 x OMe), 60.4 (OMe), 65.9 (C7), 106.1 (C10, C14), 122.5 (2 x CHpy), 125.3 (C3, C5), 128.7 (C9), 141.3 (Cpy), 143.1 (C12), 146.7 (C2, C6), 150.5 (C11, C13), 152.9 (C4), 153.0 (CHpy), 189.6 (C8); Anal. Calcd. for C21H21BrN2O4 (C, H, N): C, 56.64; H, 4.75; N, 6.29; Found: C, 56.65; H, 4.78; N, 6.26.

1-(2-oxo-2-(3,5-dimethoxyphenyl)ethyl)-[4,4'-bipyridin]-1-ium bromide (8f). Cream solid, yield 30%, mp 220-223 ºC. IR ν(cm^{-1}): 3030, 2940, 1690, 1640, 1595, 1545, 1458, 1352, 1204, 1157, 1061). ^{1}H NMR (500 MHz, DMSO-d_6): δ 3.86 (s, 6H, 2 x OMe), 6.50 s, (2H, H_7), 6.95 (bs, 1H, H_{12}), 7.20 (d, J = 1.5 Hz, 2H, H_{10}, H_{14}), 8.08 (d, J = 5.5 Hz, 2H, 2 x H_{py}), 8.75 (d, J = 6.5 Hz, 2H, H_3, H_5), 8.90 (d, J = 5.0 Hz, 2H, 2 x H_{py}), 9.13 (d, J = 6.5 Hz, 2H, H_2, H_6). ^{13}C NMR (125 MHz, DMSO-d_6): δ 55.8 2 x OMe, 66.0 C_7, 106.1 C_{10}, C_{14}, 106.2 C_{12}, 122.2 2 x CH_{py}, 125.3 C_3, C_5, 135.4 C_9, 141.2 C_{py}, 146.8 C_2, C_6, 150.8 2 x CH_{py}, 153.0 C_4, 160.9 C_{11}, C_{13}, 190.4 C_8. Anal. Calcd. for $C_{20}H_{19}BrN_2O_3$: C, 57.84; H, 4.61; N, 6.75. Found: C, 57.83; H, 4.60; N, 6.78.

1-(2-(3,4-dimethoxyphenyl)-2-oxoethyl)-[4,4'-bipyridin]-1-ium bromide (8g). Cream solid, yield 30%, mp 258-260 ºC. IR ν(cm^{-1}): 3026, 2945, 1674, 1638, 1586, 1462, 1269, 1217, 1171, 1132, 1013, 816. ^{1}H NMR (500 MHz, DMSO-d_6): δ 3.86 (s, 3H, OMe), 3.91 (s, 3H, OMe), 6.56 (s, 2H, H_7), 7.24 (d, *J* = 8.5 Hz, 1H, H_{13}), 7.54 (s, 1H, H_{10}), 7.79 (d, *J* = 8.5 Hz, 1H, H_{14}), 8.24 (bs, 2H, 2 x H_{py}), 8.78 (d, *J* = 6.0 Hz, 2H, H_3, H_5), 8.97 (bs, 2H, 2 x H_{py}), 9.20 (d, *J* = 6.0 Hz, 2H, H_2, H_6). ^{13}C NMR (125 MHz, DMSO-d_6): δ 55.8 OMe, 56.0 OMe, 65.6 C_7, 110.3 C_{10}, 111.3 C_{13}, 123.4 C_{14}, 124.4 2 x CH_{py}, 125.2 C_3, C_5, 126.5 C_9, 141.2 C_{py}, 146.8 C_2, C_6, 148.9 C_{11}, 152.9 2 x CH_{py}, 154.4 C_{12}, C_4, 188.9 C_8. Anal. Calcd. for $C_{20}H_{19}BrN_2O_3$: C, 57.84; H, 4.61; N, 6.75. Found: C, 57.83; H, 4.64; N, 6.78.

1-(2-(4-bromofenil)-2-oxoetil)-[4,4'-bipyridin]-1-ium bromide (8h). Yield 65%. All spectral data are in agreement with the literature.[2,3]

1'-(2-(3,4,5-trimethoxyphenyl)-2-oxoethyl)-[2,4'-bipyridin]-1'-ium bromide (8i). White solid, yield 64% , mp 198- 199 °C. IR ν(cm^{-1}): 3066, 3014, 2950, 2835, 1694, 1637, 1592, 1462, 1411, 1349, 1321, 1162, 1117, 992. ^{1}H NMR (500 MHz, DMSO-d$_6$): δ_{ppm} 3.81 (s, 3H, OMe), 3.91 (s, 6H, 2 x OMe), 6.54 (s, 2H, H$_7$), 7.38 (s, 2H, H$_{10}$, H$_{14}$), 7.71 (dd, J= 7.5; 4.5 Hz, 1H, H$_{py}$), 8.16 (dt, J= 8.0; 1.5 Hz, 1H, H$_{py}$), 8.49 (d, J= 7.5 Hz, 1H, H$_{py}$), 8.91 (dd, J= 5.0; 1.5 Hz, 1H, H$_{py}$), 8.93 (d, J= 7.0 Hz, 2H, H$_3$, H$_5$), 9.09 (d, J= 7.0 Hz, 2H, H$_2$, H$_6$). ^{13}C NMR (125 MHz, DMSO-d$_6$): δ 56.4 2 x OMe, 60.4 OMe, 65.8 C$_7$, 106.0 C$_{10}$, C$_{14}$, 123.7 CH$_{py}$, 124.2 C$_3$, C$_5$, 126.8 CH$_{py}$, 128.8 C$_9$, 138.4 CH$_{py}$, 143.2 C$_{12}$, 146.7 C$_2$, C$_6$, 149.8 C$_{py}$, 150.8 CH$_{py}$, 153.1 C$_{11}$, C$_{13}$, 153.6 C$_4$, 189.8 C$_8$. Anal. Calcd. for $C_{21}H_{21}BrN_2O_4$: C, 56.64; H, 4.75; N, 6.29. Found: C, 56.62; H, 4.70; N, 6.31.

1'-(2-(3,5-dimethoxyphenyl)-2-oxoethyl)-[2,4'-bipyridin]-1'-ium bromide (8j). White solid, yield 92%, mp 245-246 °C. IR ν(cm^{-1}): 3012, 2937, 1694, 1647, 1594, 1451, 1351, 1294, 1209, 1157, 1023, 847. ^{1}H NMR (500 MHz, DMSO-d$_6$): δ 3.86 (s, 6H, 2 x OMe), 6.47 (s, 2H, H$_7$), 6.95 (bs, 1H, H$_{12}$), 7.19 (d, J= 2.0 Hz, 2H, H$_{10}$, H$_{14}$), 7.71 (dd, J= 7.0; 5.0 Hz, 1H, H$_{py}$), 8.16 (dt, J= 7.5; 1.5 Hz, 1H, H$_{py}$), 8.49 (d, J= 7.5 Hz, 1H, H$_{py}$), 8.91 (dd, J= 5.0; 1.5 Hz, 1H, H$_{py}$), 8.93 (d, J= 7.0 Hz, 2H, H$_3$, H$_5$), 9.07 (d, J= 6.5 Hz, 2H, H$_2$, H$_6$). ^{13}C NMR (125 MHz, DMSO-d$_6$): δ 55.8 2 x OMe, 65.9 C$_7$, 106.1 C$_{10}$, C$_{14}$, 106.2 C$_{12}$, 123.6 C$_{20}$, 124.2 C$_3$, C$_5$, 126.8 C$_{18}$, 135.5 C$_9$, 138.4 C$_{19}$, 146.7 C$_2$, C$_6$, 149.8 C$_{15}$, 150.8 C$_{17}$, 153.6 C$_4$, 160.9 C$_{11}$, C$_{13}$, 190.6 C$_8$. Anal. Calcd. for $C_{20}H_{19}BrN_2O_3$: C, 57.84; H, 4.61; N, 6.75. Found: C, 57.80; H, 4.57; N, 6.77.

1'-(2-(3,4-dimethoxyphenyl)-2-oxoethyl)-[2,4'-bipyridin]-1'-ium bromide (8k). White solid, yield 76%, mp 263-264 °C. IR ν(cm^{-1}): 3041, 2950, 2891, 1680, 1641, 1587, 1523, 1474, 1346, 1273, 1141, 1018, 778. ^{1}H NMR (500MHz, DMSO-d$_6$): δ 3.86 (s, 3H, OMe), 3.91 (s, 3H, OMe), 6.49 (s, 2H, H$_7$), 7.24 (d, J= 8.5 Hz, 1H, H$_{10}$), 7.53 (d, J= 2.0 Hz, 1H, H$_{13}$), 7.53 (d, J= 8.5 Hz, 1H, H$_{13}$), 7.71 (dd, J= 7.5; 5.0 Hz, 1H, H$_{py}$), 7.78 (dd, J= 8.5; 2.0 Hz, 1H, H$_{14}$), 8.16 (dt, J= 8.0; 2.0 Hz, 1H, H$_{py}$), 8.49 (d, J= 8.0 Hz, 1H, H$_{py}$), 8.91 (dd, J= 5.0; 2.0 Hz, 1H, H$_{py}$), 8.92 (d, J= 7.0 Hz,

2H, H_3, H_5), 9.10 (d, J= 6.5 Hz, 2H, H_2, H_6). ^{13}C-NMR (125 MHz, DMSO-d_6): δ 55.8 OMe, 56.0 OMe, 65.5 C_7, 110.3 C_{10}, 111.3 C_{13}, 123.4 CH_{py}, 123.6 C_{14}, 124.1 C_3, C_5, 126.3 C_9, 126.8 CH_{py}, 138.3 CH_{py}, 146.8 C_2, C_6, 148.9 C_{11}, 149.8 C_{py}, 150.8 CH_{py}, 154.3 C_{12}, 153.5 C_4, 189.1 C_8. Anal. Calcd. for $C_{20}H_{19}BrN_2O_3$: C, 57.84; H, 4.61; N, 6.75. Found: C, 57.82; H, 4.60; N, 6.79.

1'-(2-(4-bromophenyl)-2-oxoethyl)-[2,4'-bipyridin]-1'-ium bromide (8l). Cream solid, yield 94%, mp 253-255 ^{o}C. IR ν(cm^{-1}): 2987, 2936, 1693, 1636, 1578, 1341, 988, 775.^{1}H NMR (500MHz, DMSO-d_6): δ 6.47 (s, 2H, H_7), 7.71 (dd, J= 8.0; 5.5 Hz, 1H, H_{py}), 7.92 (d, J= 8.5 Hz, 2H, H_{11}, H_{13}), 8.02 (d, J= 8.5 Hz, 2H, H_{10}, H_{14}), 8.16 (dt, J= 8.0; 1.5 Hz, 1H, H_{py}), 8.48 (d, J= 8.0 Hz, 1H, H_{py}), 8.91 (dd, J= 5.5; 1.5 Hz, 1H, H_{py}), 8.93 (d, J= 7.0 Hz, 2H, H_3, H_5), 9.07 (d, J= 7.0 Hz, 2H, H_2, H_6). ^{13}C-NMR (125 MHz, DMSO-d_6): δ 65.7 C_7, 123.6 CH_{py}, 124.2 C_3, C_5, 126.8 CH_{py}, 128.9 C_{12}, 130.2 C_{10}, C_{14}, 132.3 C_{11}, C_{13}, 132.7 C_9, 138.4 CH_{py}, 146.8 C_2, C_6, 149.8 C_{py}, 150.8 CH_{py}, 153.6 C_4, 190.2 C_8. Anal. Calcd. for $C_{18}H_{14}Br_2N_2O$: C, 49.80; H, 3.25; N, 6.45. Found: C, 49.82; H, 3.20; N, 6.49.

1-(2-oxo-2-(3,4,5-trimethoxyphenyl)ethyl)-[2,2'-bipyridin]-1-ium bromide (13a). Cream solid, yield 65%, mp 197-199 °C. IR ν(cm^{-1}): 3057, 3016, 2977, 2945, 1692, 1583, 1416, 1318, 1161, 1124, 991. ^{1}H NMR (500MHz, DMSO-d_6): δ 3.78 (s, 3H, OMe), 3.84 (s, 6H, 2 x OMe), 6.53 (s, 2H, H_7), 7.26 (s, 2H, H_{10}, H_{14}), 7.62 (ddd, J= 7.5; 5.0; 1.0 Hz, 1H, H_{18}), 8.10 (d, J= 7.5 Hz, 1H, H_{20}), 8.15 (dt, J= 8.0; 1.5 Hz, 1H, H_{19}), 8.39-8.42 (overlapped signals, 2H, H_{17}, H_5), 8.55 (dd, J= 8.0; 1.0 Hz, 1H, H_3), 8.90 (dt, J= 7.5; 1.0 Hz, 1H, H_4), 9.23 (dd, J= 6.0; 1.0 Hz, 1H, H_6). ^{13}C NMR (125 MHz, DMSO-d_6): δ 56.3 2 x OMe, 60.3 OMe, 64.6 C_7, 106.0 C_{10}, C_{14}, 126.1 C_{18}, 126.7 C_{20}, 127.5 C_5, 128.6 C_9, 130.1 C_3, 138.6 C_{19}, 142.8 C_{12}, 147.2 C_{15}, 148.9 C_4, 149.0 C_6, 149.1 C_{17}, 151.3 C_2, 153.0 C_{11}, C_{13}, 189.3 C_8. Anal. Calcd. for $C_{21}H_{21}BrN_2O_4$: C, 56.64; H, 4.75; N, 6.29. Found: C, 56.61; H, 4.72; N, 6.33.

1-(2-(3,5-dimethoxyphenyl)-2-oxoethyl)-[2,2'-bipyridin]-1-ium bromide (13b). Cream solid, yield 22%, mp 195-196 °C. IR ν(cm^{-1}): 3057, 3027, 2988, 2973, 1687, 1592, 1443, 1294, 1200, 1150, 1021, 765. ^{1}H NMR (500MHz, DMSO-d_6): δ 3.82 (s, 6H, 2 x OMe), 6.40 (s, 2H, H_7),

6.89 (t, J= 2.0 Hz, 1H, H_{12}), 7.26 (d, J= 2.0 Hz, 2H, H_{10}, H_{14}), 7.61 (ddd, J= 7.5; 4.5; 1.0 Hz, 1H, H_{18}), 8.09 (d, J= 8.0 Hz, 1H, H_{20}), 8.16 (dt, J= 8.0; 1.5 Hz, 1H, H_{19}), 8.38 (dd, J= 6.5; 1.5 Hz, 1H, H_{17}), 8.39 (dt, J= 7.5; 1.5 Hz, 1H, H_5), 8.54 (dd, J= 8.0; 1.0 Hz, 2H, H_3), 8.89 (dt, J= 8.0; 1.5 Hz, 1H, H_4), 9.16 (dd, J= 6.0; 1.0 Hz, 1H, H_6). ^{13}C NMR (125 MHz, DMSO-d_6): δ 55.7 2 x OMe, 64.8 C_7, 105.9 C_{10}, C_{14}, C_{12}, 126.1 C_{18}, 126.7 C_{20}, 127.5 C_5, 130.0 C_3, 135.3 C_9, 138.6 C_{19}, 147.2 C_4, 148.9 C_6,149.0 C_{17}, C_{15}, 151.2 C_2, 160.8 C_{11}, C_{13}, 189.9 C_8. Anal. Calcd. for $C_{20}H_{19}BrN_2O_3$: C, 57.84; H, 4.61; N, 6.75. Found: C, 57.81; H, 4.56; N, 6.78.

1-(2-(3,4-dimethoxyphenyl)-2-oxoethyl)-[2,2'-bipyridin]-1-ium bromide (13c). Cream solid, yield 33%, mp 199-201 °C. IR ν(cm^{-1}): 3060, 3032, 2979, 1686, 1583, 1515, 1419, 1270, 1165, 1151, 1012, 770. ^{1}H NMR (500MHz, DMSO-d_6): δ 3.81 (s, 3H, OMe), 3.88 (s, 3H, OMe), 6.43 (s, 2H, H_7), 7.14 (d, J= 8.5 Hz, 1H, H_{13}), 7.43 (d, J= 1.5 Hz, 1H, H_{10}), 7.60 (dd, J= 7.0; 5.0 Hz, 1H, H_{18}), 7.64 (dd, J= 8.5; 1.5 Hz, 1H, H_{14}), 8.06 (d, J= 8.0 Hz, 1H, H_{20}), 8.14 (dt, J= 8.0; 1.0 Hz, 1H, H_{19}), 8.37-8.40 (overlapped signals, 2H, H_{17}, H_5), 8.52 (d, J= 8.0 Hz, 2H, H_3), 8.88 (t, J= 8.0 Hz, 1H, H_4), 9.19 (d, J= 6.0 Hz, 1H, H_6). ^{13}C NMR (125 MHz, DMSO-d_6): δ 55.7 OMe, 56.0 OMe, 66.3 C_7, 110.2 C_{10}, 111.2 C_{13}, 123.1 C_{14}, 126.0 C_9, 126.1 C_{18}, 126.7 C_{20}, 127.4 C_5, 130.0 C_3, 138.6 C_{19}, 147.1 C_4, 148.9 C_{11}, 149.0 C_6, C_{17}, 149.2 C_{15}, 151.4 C_2, 154.1 C_{12}, 188.6 C_8. Anal. Calcd. for $C_{20}H_{19}BrN_2O_3$: C, 57.84; H, 4.61; N, 6.75. Found: C, 57.80; H, 4.57; N, 6.79.

1-(2-(3-bromophenyl)-2-oxoethyl)-[2,2'-bipyridin]-1-ium bromide (13d). Cream solid, yield 20%, mp 207-210°C. IR ν(cm^{-1}) 3055, 3018, 2979, 2919, 1704, 1583, 1442, 1225, 992, 774. ^{1}H NMR (500MHz, DMSO-d_6): δ 6.40 (s, 2H, H_7), 7.59 (dd, J= 7.5; 5.5 Hz, 1H, H_{18}), 7.85 (d, J= 8.5 Hz, 2H, H_{11}, H_{13}), 7.93 (d, J= 8.5 Hz, 2H, H_{10}, H_{14}), 8.10 (d, J= 8.0 Hz, 1H, H_{20}), 8.16 (t, J= 8.0 Hz, 1H, H_{19}), 8.26 (d, J= 4.5 Hz, 1H, H_{17}), 8.40 (t, J= 6.5 Hz, 1H, H_5), 8.54 (d, J= 7.5 Hz, 2H, H_3), 8.89 (t, J= 8.0 Hz, 1H, H_4), 9.17 (d, J= 6.0 Hz, 1H, H_6). ^{13}C NMR (125 MHz, DMSO-d_6): δ 64.5 C_7, 126.2 C_{18}, 126.8 C_{20}, 127.5 C_5, 128.6 C_{12}, 130.0 C_3, 130.1 C_{10}, C_{14}, 132.3 C_{11}, C_{13}, 132.5 C_9, 138.7 C_{19}, 147.2 C_4, 148.9 C_{17}, 149.0 C_{15}, 149.1 C_6, 151.0 C_2, 189.6 C_8. Anal. Calcd. for $C_{18}H_{14}Br_2N_2O$: C, 49.80; H, 3.25; N, 6.45. Found: C, 49.83; H, 3.21; N, 6.48.

References

1. Lucescu L, Ghinet A, Belei D, Rigo B, Dubois J, Bicu E. Discovery of indolizines containing triazine moiety as new leads for the development of antitumoral agents targeting mitotic events. Bioorg Med Chem Lett. 2015;25:3975-3979
2. Pricope G, Ursu EL, Sardaru M, Cojocaru C, Clima L, Marangoci N, Danac R, Mangalagiu I, Simionescu BC, Pinteala M, et.al. Novel cyclodextrin-based pH-sensitive supramolecular host-guest assembly for staining acidic cellular organelles. Polym Chem. 2018;9:968-975.
3. Rotaru A, Druta I, Oeser T, Müller TJJ. A novel coupling 1,3- dipolar cycloaddition sequence as a three- component approach to highly fluorescent indolizines. Helv Chim Acta. 2005;88:1798-1812.

^{1}H NMR and ^{13}C NMR spectra of compound **11a**

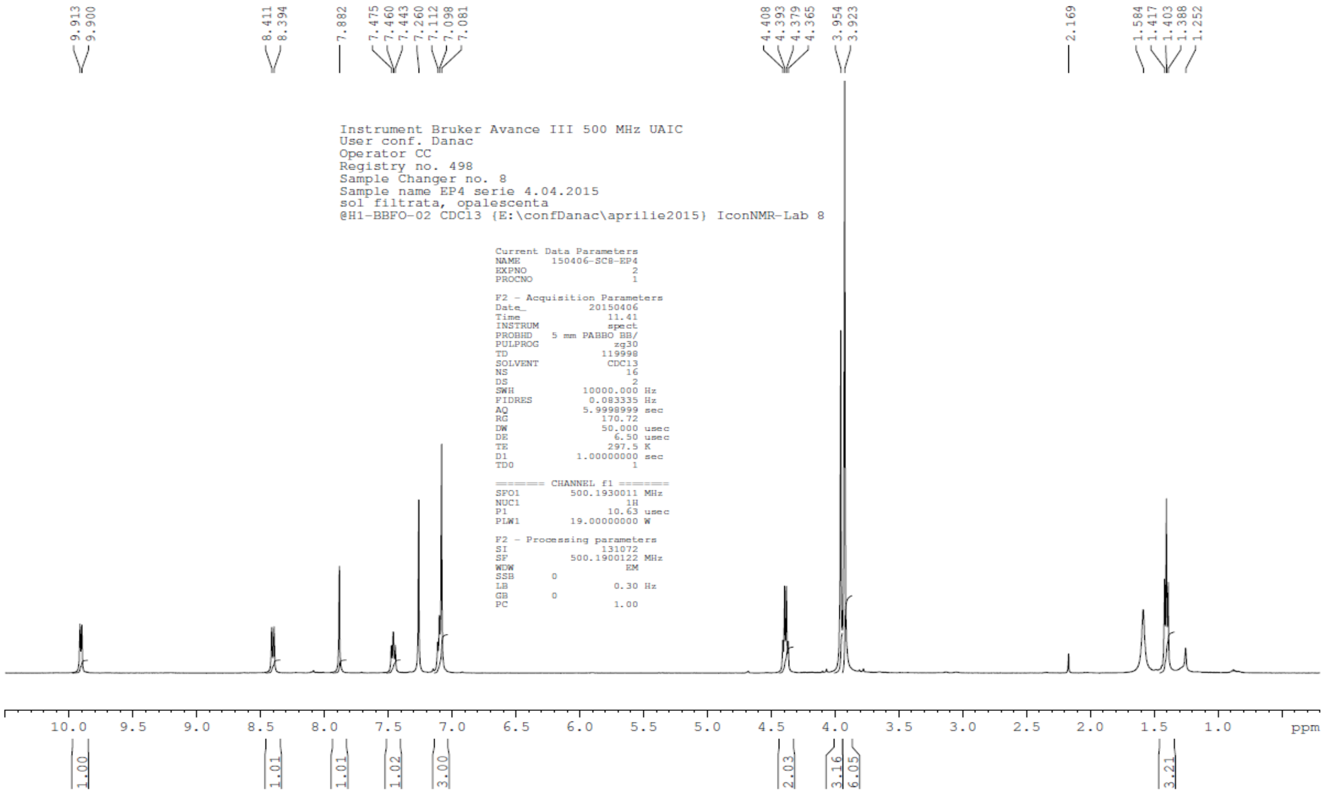

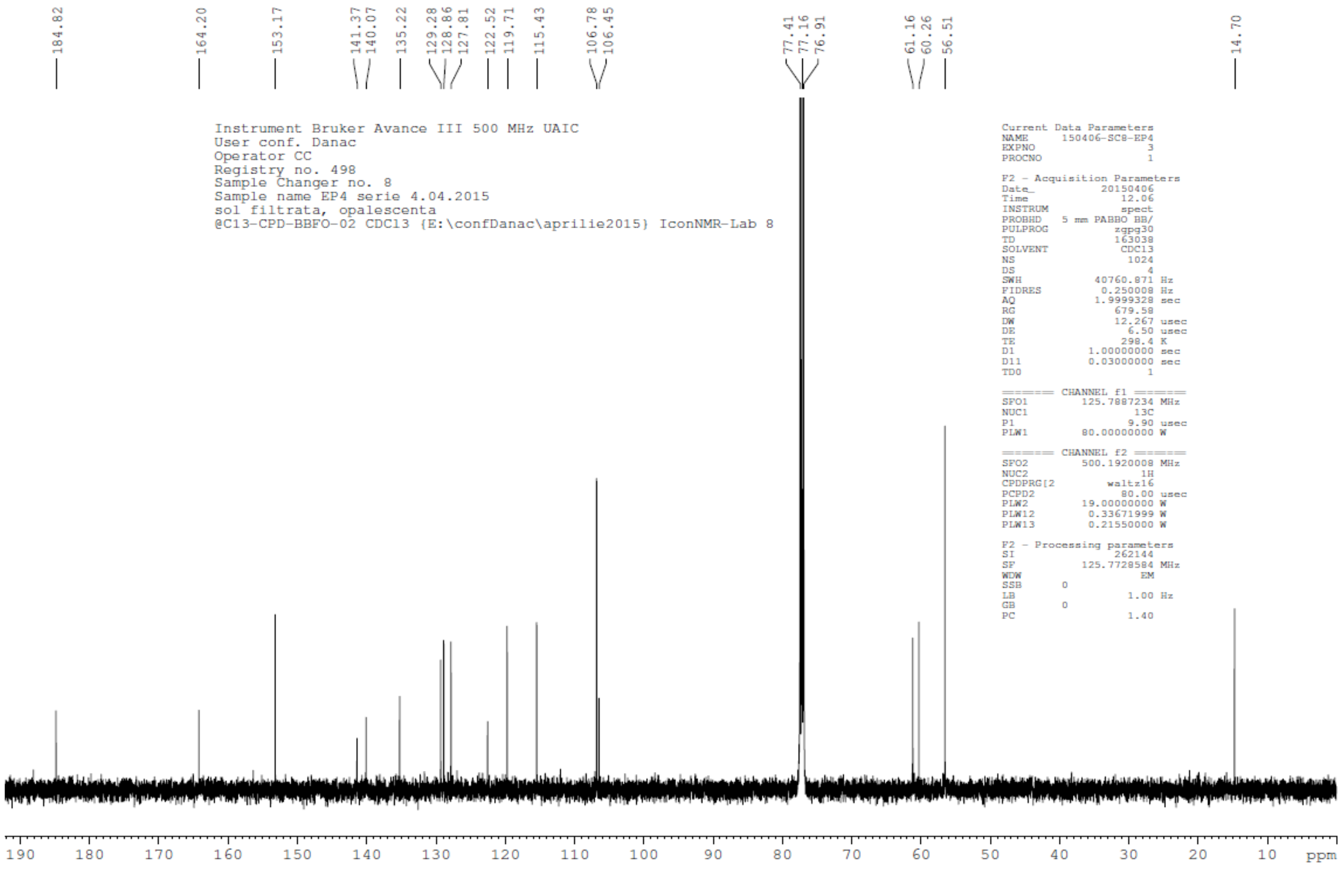

^{1}H NMR and ^{13}C NMR spectra of compound **11b**

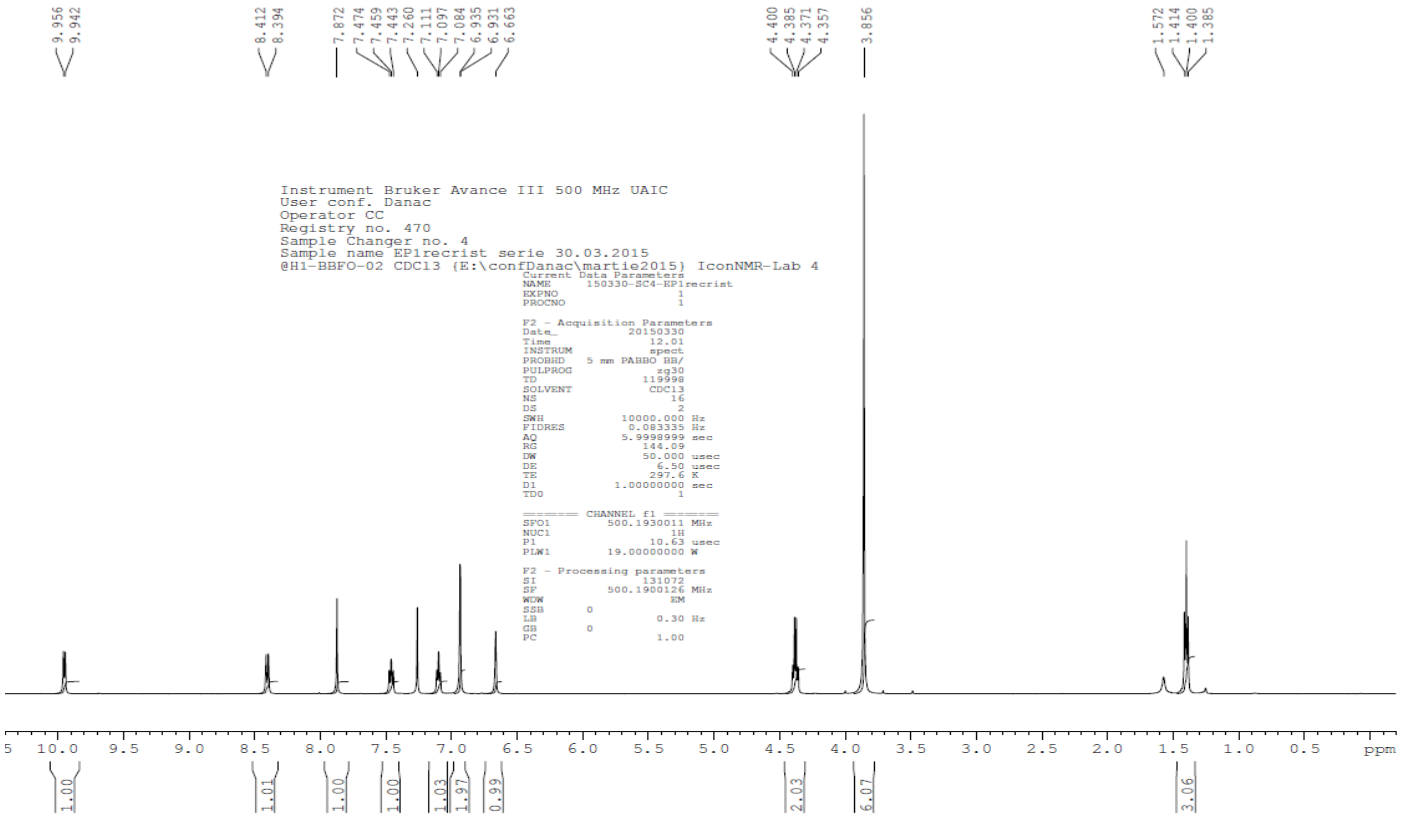

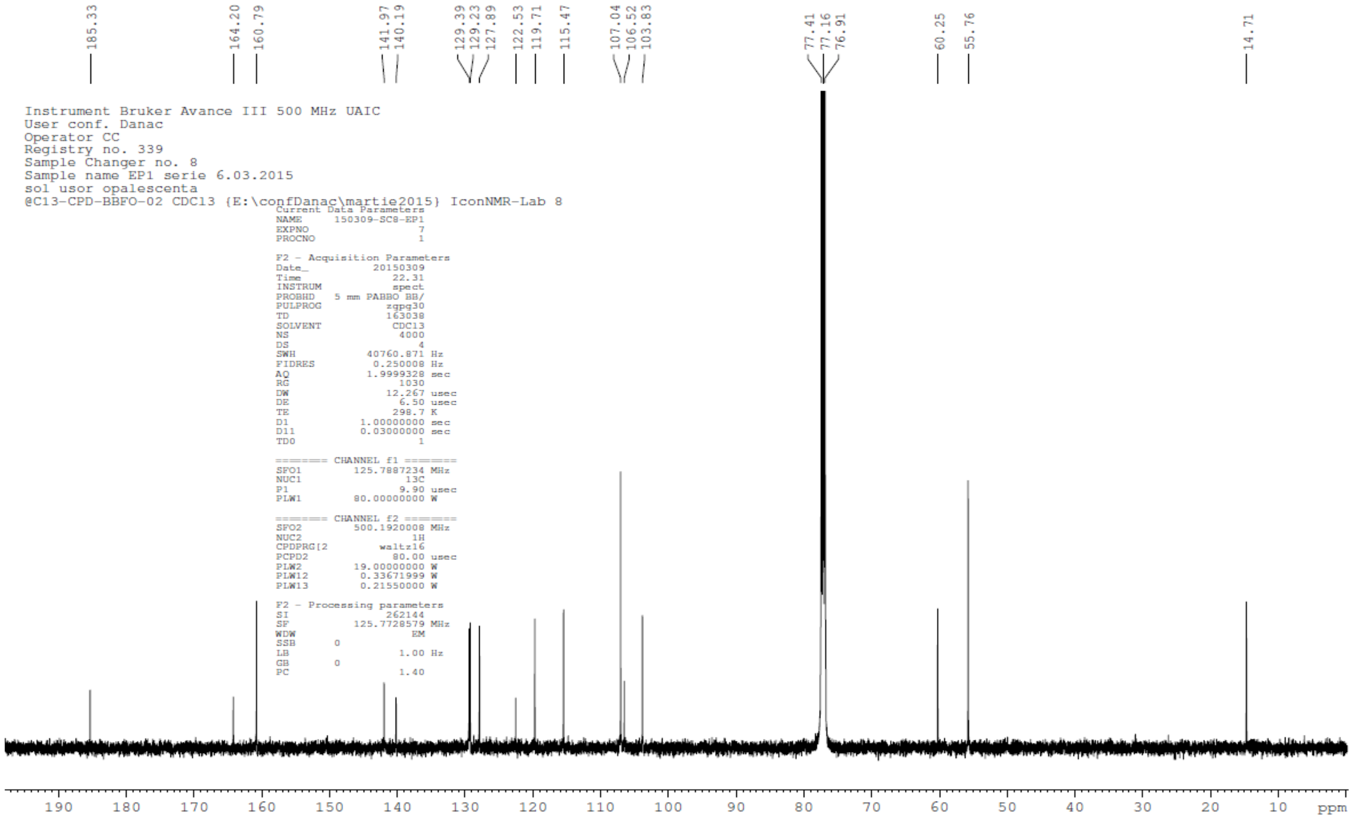

^{1}H NMR and ^{13}C NMR spectra of compound **11k**

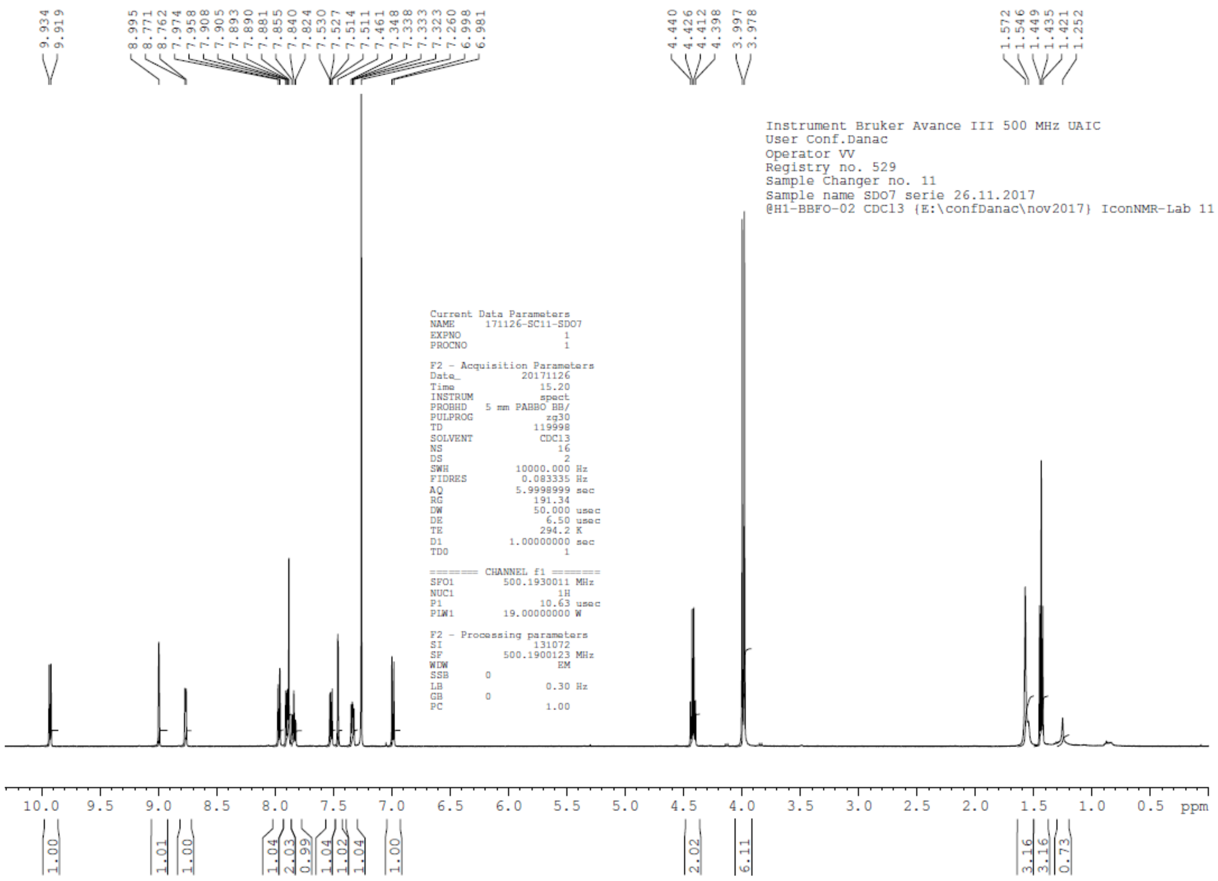

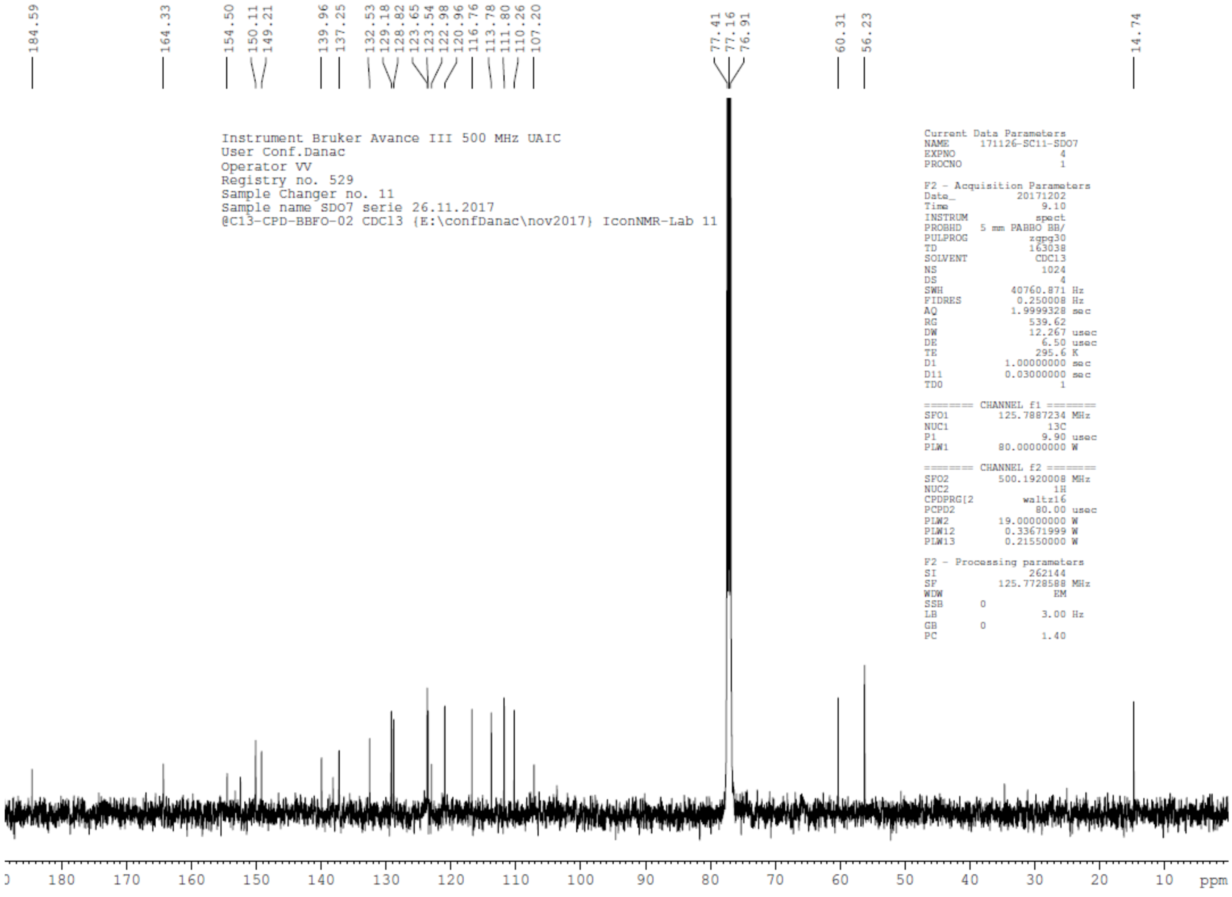

^{1}H NMR and ^{13}C NMR spectra of compound **14a**

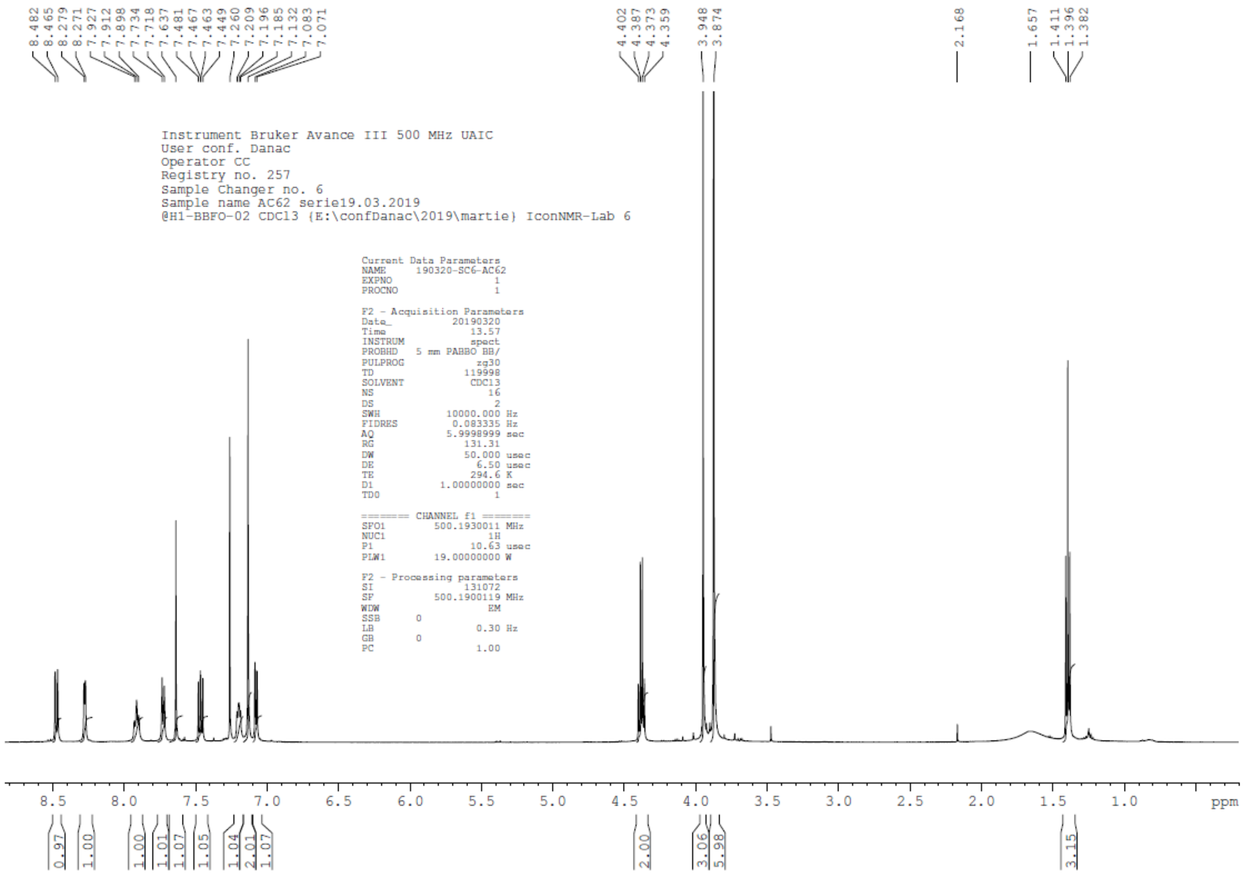

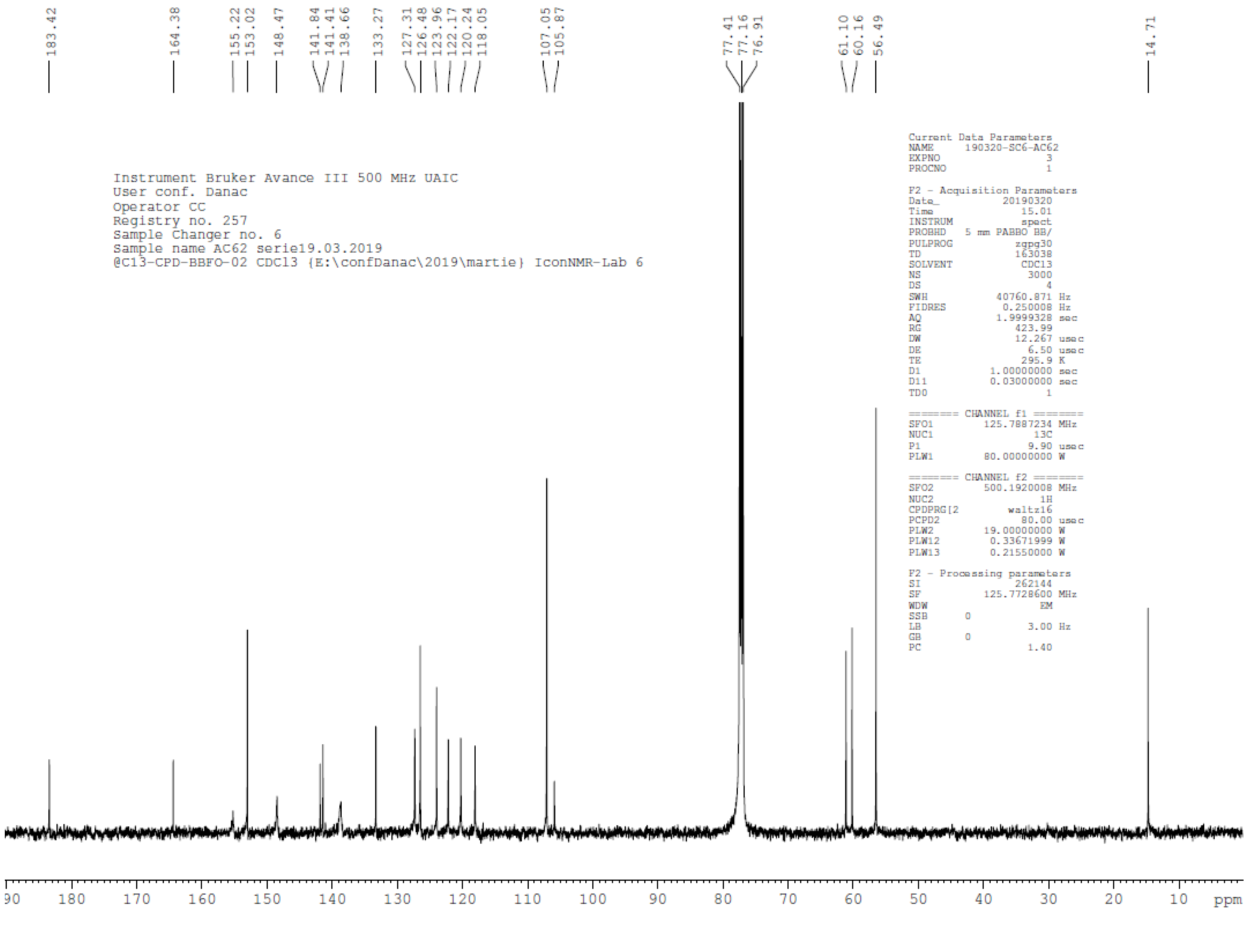

^{1}H NMR and ^{13}C NMR spectra of compound **15a**

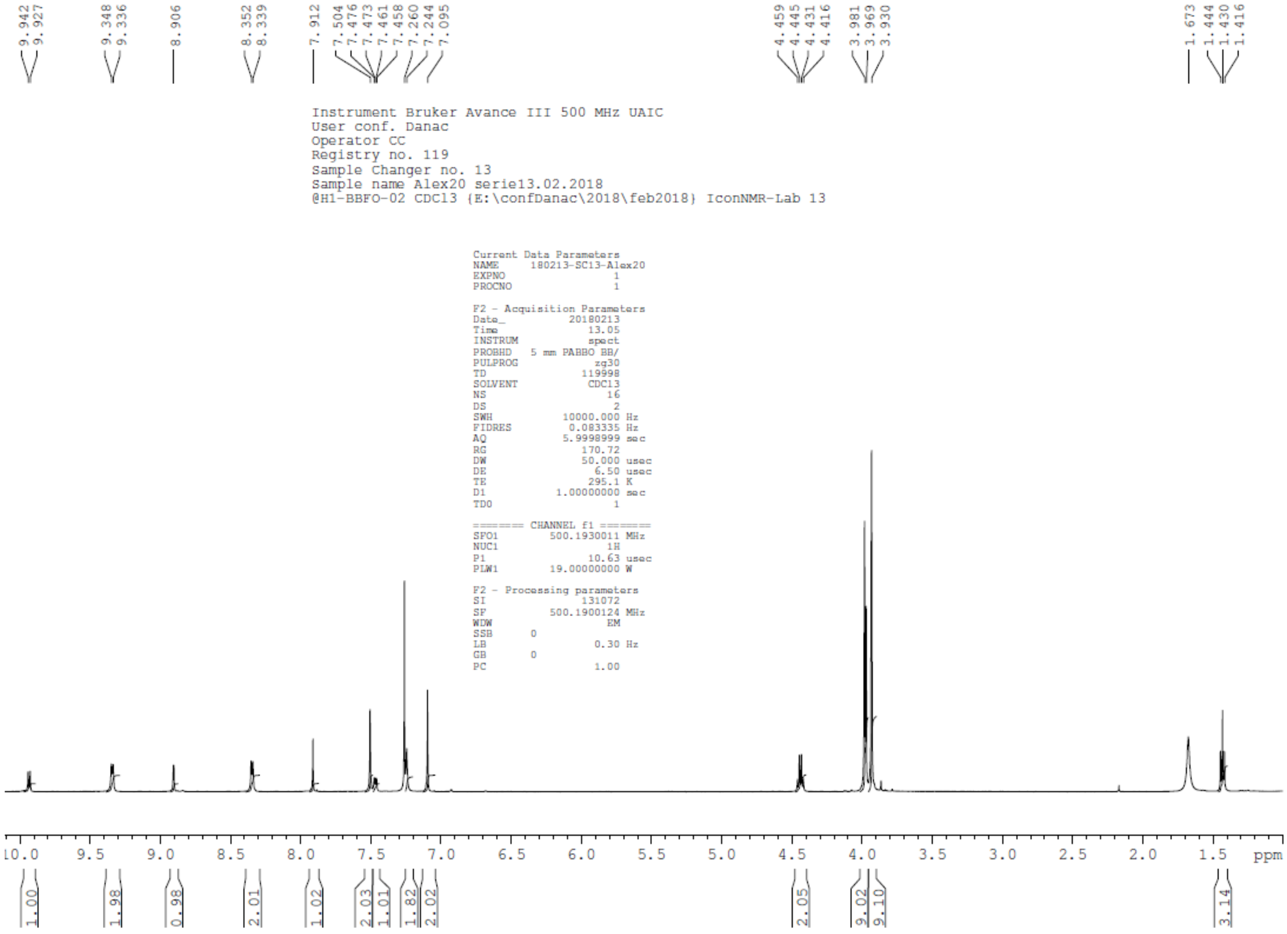

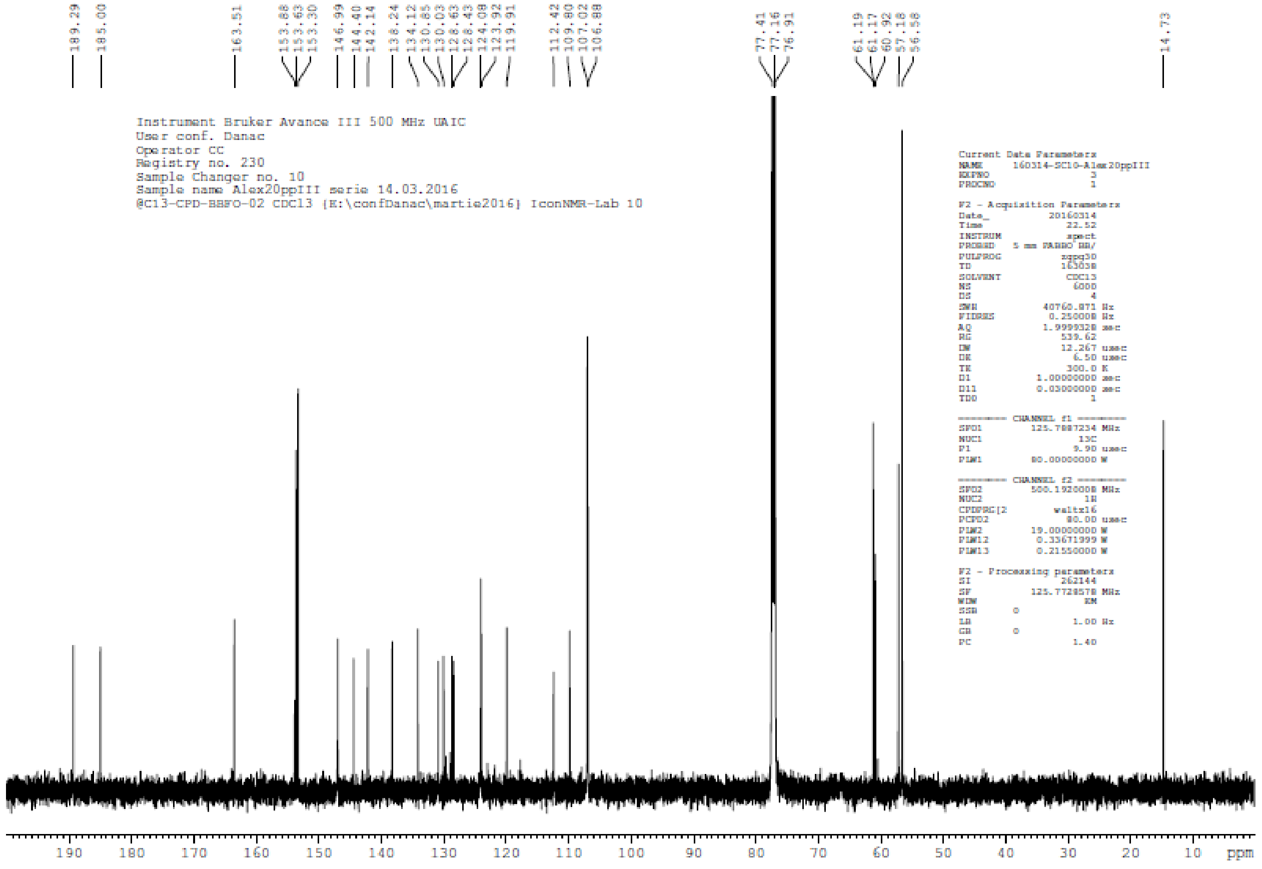

^{1}H NMR and ^{13}C NMR spectra of compound **15j**

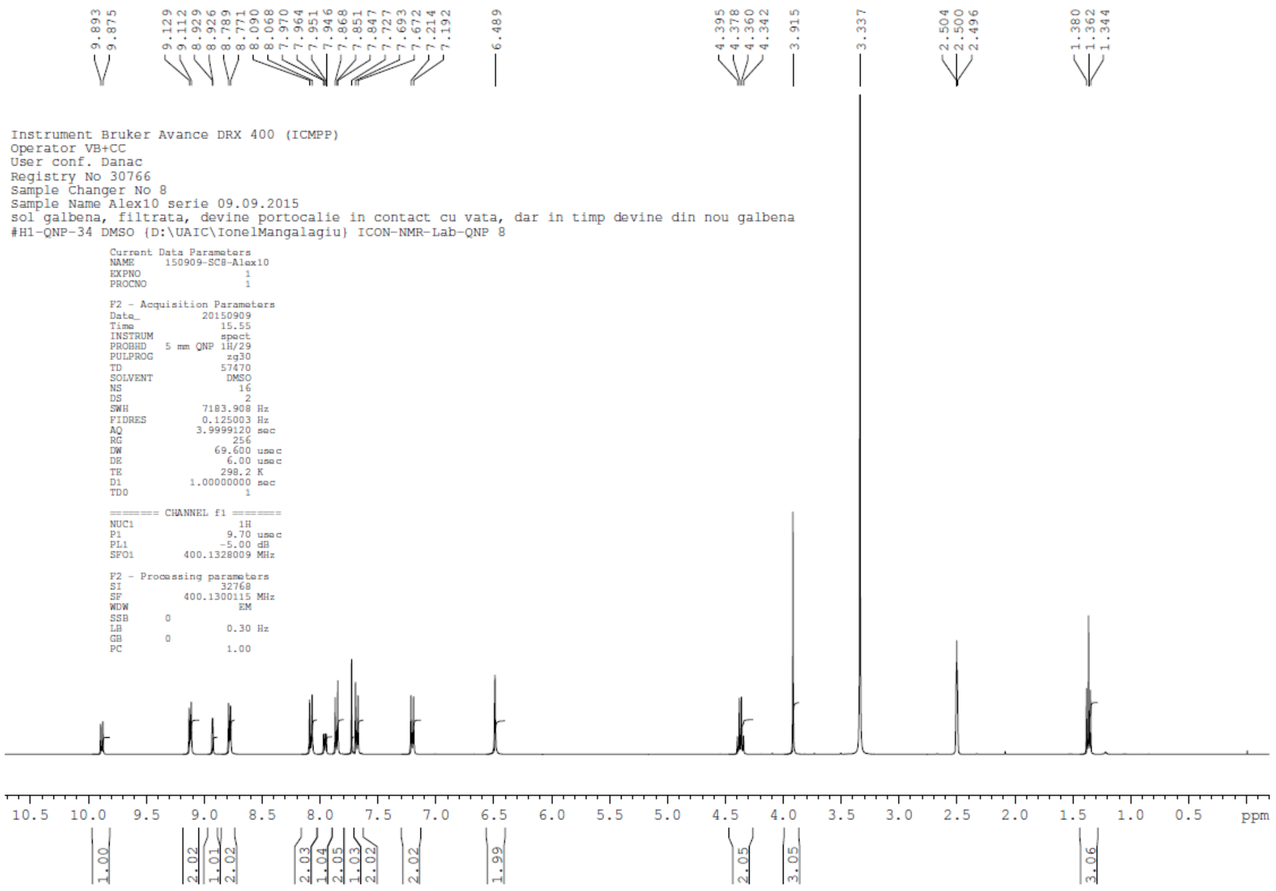

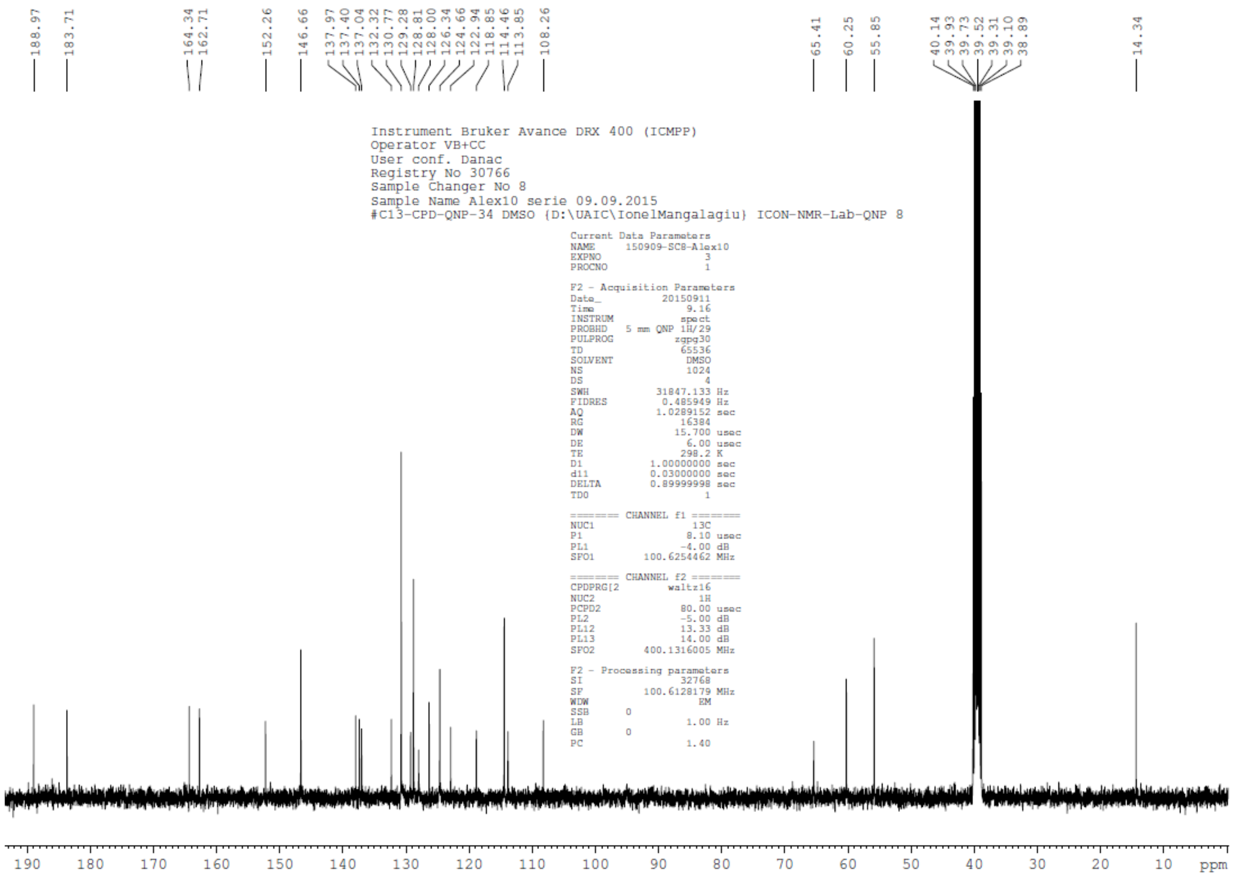

Supplement: Supplemental Material [file IENZ_A_1801671_SM1921.pdf]
